# Multiple-context extinction strengthens fear suppression through hippocampal dopamine D2R-dependent memory updating

**DOI:** 10.1038/s41386-026-02577-x

**Published:** 2026-10-06

**Authors:** Eve A. Hammerman, Suzanne R. Penna, Shania E. Gonzalez, Shiv Patel, Carter Roebuck, Emily K. Lock, Cooper Desmond, Yuichi Fukunaga, Gwyneth C. Maloy, Victor A. Cazares

**Affiliations:** 1 Psychology Department, Neuroscience Program, Williams College, Williamstown, MA, USA.; 2 These authors contributed equally: Suzanne R. Penna, Shania E. Gonzalez.

## Abstract

Extinction learning, which underlies exposure therapy, generates safety memories that are often context-dependent, limiting their persistence and generalization. Although varying contextual cues during extinction has been proposed to strengthen safety learning, the neural mechanisms remain unknown. Here, we compared fear extinction conducted in a single context (SCFE) or multiple contexts (MCFE) in 129S1 and C57BL/6J mice. MCFE enhanced extinction learning and generalized extinction recall in novel contexts at 2-d, with this advantage persisting at 30-d when intervening testing occurred in the trained extinction context. This enhancement required exposure to distinct, but not novel, contexts and did not prevent spontaneous recovery or US-induced reinstatement. Dual Fos-TRAP activity mapping revealed differential recruitment of the fear conditioning-tagged cells following MCFE and identified the hippocampus as a key node supporting MCFE. Consistent with this, chemogenetic silencing of dorsal, but not ventral, hippocampus selectively impaired extinction acquisition during MCFE and abolished its behavioral advantage. Likewise, local blockade of dopamine D2-, but not D1-like, receptors in the dorsal hippocampus eliminated the enhancement in extinction acquisition produced by MCFE. Together, these findings demonstrate that contextual variation strengthens and generalizes extinction learning through dorsal hippocampal dopamine D2-like receptor-dependent memory updating, identifying a neural mechanism through which behavioral modifications of exposure therapy can improve fear suppression.

## INTRODUCTION

Exposure therapy is the first-line behavioral treatment for PTSD and other fear-related disorders, yet fear relapse occurs in up to 50% of patients [1]. This high relapse rate is thought to reflect the failure of extinction-based safety memories to generalize across contexts and endure over time [2–4].

Exposure therapy is based on fear extinction (FE), the learning process in which repeated, safe encounters with feared cues reduce maladaptive fear and avoidance. Experimentally, FE is accomplished by repeatedly presenting a conditioned stimulus (CS; e.g., tone) in the absence of the aversive unconditioned stimulus (US; e.g., footshock) that it previously signaled, resulting in progressive reduction of conditioned fear [5]. Importantly, extinction does not erase the original fear memory. Instead, it establishes a new safety memory that competes with the fear memory for behavioral expression [6]. Consequently, extinguished fear can return after the passage of time (spontaneous recovery), when the CS is encountered in a new context (fear renewal), or when the aversive US is reexperienced (fear reinstatement). Once a CS predicts both threat and safety, contextual cues become necessary for resolving this ambiguity by determining which memory is retrieved.

The context-dependence of cued extinction is evident not only behaviorally but also in the neural representations of fear and safety memories. In rodents, neural ensembles encoding learned fear are readily reactivated outside the extinction context, whereas extinction-related ensembles remain tightly linked to the context in which they were acquired [7, 8]. Human functional neuroimaging studies similarly demonstrate that neural representations of aversive memories resemble one another regardless of context or type of threat, whereas extinction produces distinct, context-specific representations [9–11]. Together, these findings suggest that reducing the contextual dependence of extinction memories may improve their generalizability.

One promising strategy is to vary the contexts in which extinction occurs. While contextual variation can promote acute fear relapse through renewal, when incorporated into extinction training, it may paradoxically promote extinction memory updating and more generalizable attenuation of learned fear [12–20], *cf* [19, 20]. Indeed, emerging clinical recommendations emphasize varying internal, external, and interpersonal contexts to enhance retrieval and generalization of safety learning [21–27]. Such effects may arise in part from associative novelty, whereby changes in learned stimulus-context relationships generate expectancy violations that promote memory updating [28, 29]. Recent work has implicated dopamine in extinction updating, but whether it supports updating driven by changes in CS-context relationship remains unknown [30–32].

Here, we establish and validate a multiple-context fear extinction (MCFE) paradigm that reliably produces more effective and generalized extinction learning than conventional single-context extinction. We further identify dorsal hippocampal dopamine D2 receptor-signaling as a critical mechanism underlying this behavioral enhancement, providing new insight into how contextual variation promotes more generalized safety learning.

## METHODS AND MATERIALS

### Animals

Founder mice were sourced from Jackson Laboratories and bred in-house. Strain selection varied by experiment according to the experimental objective (see Supplementary Methods). Mice were group-housed with *ad libitum* food and water access on a 12-h light/dark cycle, with all experiments conducted during the light phase. All procedures using mice were approved by the Williams College Institutional Care and Use Committee.

### Behavior

Behavioral assays were conducted in fear conditioning chambers (#VFC2-USB-M, Med-Associates). Behavior was recorded at 30 Hz using front-facing cameras with near-infrared pass filters. *VideoFreeze* (Med-Associates) was used for stimulus delivery and video acquisition. Behavioral assessments were conducted as follows:

- Fear conditioning (FC; days 1–2): Three CS-tone presentations (4 kHz, 25-sec), each immediately followed by a 2-sec 0.7–0.75 mA footshock (US) in context A.
- Fear extinction (FE, days 3–5): Twelve unreinforced CS-tone presentations. For single-context fear extinction (SCFE), all three training sessions (FE1-FE3) were conducted in context B. For MCFE, extinction sessions were conducted in distinct contexts (B–D).
- Recall and reinstatement testing: In selected experiments, extinction recall was assessed 2 and 30 days following FE using 4 unreinforced CS-tones. For reinstatement, mice were re-exposed to three unsignaled footshocks in Context A following extinction training.

Distinct behavioral contexts were established by varying the chamber walls, flooring, ambient odors, and lighting conditions (Supplementary Table 1).

### Dual labeling: Fos neuronal tagging and immunofluorescence

Dual Fos-labeling of active neuronal populations was achieved by combining TRAP2 (Targeted Recombination of Active Populations 2) and Fosimmunolabeling [33]. Activity-dependent neuronal tagging was induced by a single intraperitoneal injection of tamoxifen (B5965–5000, APExBIO; 50 mg/kg) immediately following FC2. Following a 7-d delay period to permit tdTomato expression, mice underwent extinction training. Neurons active during FE3 were labeled via Fos immunohistochemistry using a rabbit anti-Fos antibody (Cell Signaling #2250). Fluorescence images were acquired using a VS200 epifluorescence microscope (Olympus).

Brain sections were aligned to the Allen Mouse Brain Common Coordinate Framework using DeepSlice [34]. TRAP- and Fos-positive cells were detected using StarDist, an automated deep-learning-based method, via the QuPath software environment [35]. Area-weighted cell densities were calculated by summing positive cell counts across sampled sections and dividing by the total annotated area for each region.

To estimate region-specific FC vs. FE recruitment bias, Fos and TRAP densities were normalized to the corresponding mean density in the US-control group for each brain region, and marker-specific recruitment was calculated as the log_10_ fold-change relative to US- controls. Regional covariance matrices were constructed from inter-regional Spearman correlations (ρ), and adjacency networks were used to visualize reorganization of extinction-related (Fos+) and reactivated fear (TRAP+ and Fos+) ensembles, defined as Δρ = ρMCFE − ρSCFE. Anatomical systems-level correlations were evaluated using animal-level bootstrapping to generate 95% confidence intervals and within-group permutation testing against a null model.

### Chemogenetics

Mice underwent stereotaxic surgery to receive bilateral injections of purified adeno-associated virus expressing either an inhibitory DREADD (AAV5-CaMKII-hM4D(Gi)-IRES-mCitrine), or a control fluorescent marker (AAV5-CaMKII-mCherry) into either the dorsal (AP −1.9; ML+/− 1.5; DV −1.8; 500 nl) or ventral (AP −3.16; ML+/−3.0; DV −3.85, −3.5, −3.25; 200 nl/site) hippocampus. Mice were habituated to handling for 7 days prior to behavioral testing. On FE2, 30 min before training, all mice received an intraperitoneal (*i.p*.) injection of the DREADD agonist Compound 21 (C21; 1 mg/kg) [36].

### Drug administration

Mice received *i.p*. or intracerebral (*i.c*.) infusions of SCH23390, raclopride, or vehicle (saline) 10–30 min prior to the FE2 behavioral session. *i.c*. infusions were delivered through bilateral 33-ga internal cannulae (PlasticsOne) at 0.2 μL/min using a dual-channel syringe pump (Conduct Science). Internal cannulae extended 0.5 mm below bilateral 26-ga guide cannulae targeting either dorsal hippocampus (AP −1.9; ML+/−1.5; DV −1.3) or infralimbic cortex (AP + 1.8; ML +/−0.25; DV −2.4). Mice were habituated to handling for 7 days prior to behavioral testing. Infusion targeting was validated via visualization of a fluorescently-labeled cholera toxin B (CTB-488, Invitrogen #C34775), and dorsal hippocampus infusate spread was estimated via visualization of sodium fluorescein (Supplementary Fig. S9).

### Statistical analyses

Behavioral outcomes were analyzed using linear mixed-effects models that included a random intercept for subject (Animal) and fixed factors matched to each design (e.g., Group, CS, Day, and their interactions) [37]. When interactions were significant, Holm-adjusted simple-effects comparisons were performed. Main-effect post-hoc tests were conducted only when no higher-order interaction involving that factor was significant. Unless noted, tests were two-tailed with α = 0.05. Where reported, standardized effect size g was calculated as the specified model contrast divided by the model’s residual standard deviation. Complete statistical results are available in Supplementary Statistics File.

## RESULTS

### Training in multiple contexts enhances extinction learning without requiring contextual novelty

To determine whether MCFE enhances extinction learning, we compared the efficacy of FE training in single vs. multiple contexts using two mouse strains that differ in stress susceptibility: the stress-susceptible 129S1 (S1) and the more stress-resilient strain C57BL/6J (B6) [38, 39]. Mice underwent two sessions of FC followed by three extinction sessions conducted either in the same context (B,B,B; SCFE) or distinct contexts (B,C,D; MCFE, Fig. 1A).

All mice acquired conditioned fear, exhibiting progressively greater freezing across CS presentations (F[2,410] = 71.50, *p* < 0.0001) and conditioning days (F[2,410] = 307.26, *p* < 0.0001) (Fig. 1B, Supplementary Fig. S1). Importantly, freezing during the first three CS presentations of the initial extinction session did not differ between groups (Fig. 1C), indicating equivalent fear recall before extinction.

We next compared extinction learning within and across training sessions. Within-session extinction (Fig. 1D) differed between strains: B6 mice exhibited progressive reductions in freezing, particularly during FE2 and FE3, whereas S1 mice showed no within-session extinction, consistent with previous reports [31]. In contrast, both strains exhibited robust between-session extinction (Fig. 1E), with freezing decreasing across days (F[2,80] = 64.71, *p* < 0.0001). However, MCFE produced significantly greater between-session extinction than SCFE, with MCFE mice exhibiting lower freezing than SCFE mice on FE3 in both strains (S1: *t*[79] = 2.57, *p* = 0.012; B6: *t*[79] = 2.04, *p* = 0.044). This greater reduction in freezing was also reflected in larger standardized FE1 → FE3 effect sizes for MCFE relative to SCFE (Fig. 1F).

Interestingly, although MCFE enhanced between-session extinction in both strains, their extinction learning trajectories differed. Whereas B6 mice exhibited relatively stable freezing between FE1 and FE2, S1 mice displayed a transient increase in freezing following the first contextual shift (B → C; *t*[80] = − 3.42, *p* < 0.0001), consistent with fear renewal. Nonetheless, freezing markedly declined by FE3, indicating that additional MCFE training overcame this renewal effect. Together, these findings demonstrate that conducting extinction training across multiple contexts enhances between-session extinction learning in both stress-susceptible and stress-resilient mouse strains.

Previous studies have shown that novel experiences can enhance memory encoding and persistence, raising the possibility that the improved extinction observed with MCFE simply reflects repeated exposure to novel environments [40–42]. To determine whether the behavioral advantage of MCFE depends on contextual novelty, we compared SCFE with extinction conducted across either multiple *familiar* (F-MCFE) or *novel* contexts (N-MCFE) in S1 mice. The F-MCFE group was pre-exposed to each extinction context 24-h before training, whereas the N-MCFE group was pre-exposed to an unrelated context, preserving the novelty of each extinction environment (Fig. 1G). SCFE mice were pre-exposed to the same extinction context (B) before each extinction training session.

All animals demonstrated robust fear acquisition, exhibiting increased freezing across CS presentations and conditioning days, with no differences among experimental groups (Supplementary Fig. S2).

Whereas SCFE animals did not exhibit reductions in freezing across extinction sessions, both F-MCFE and N-MCFE animals showed significant reductions in freezing from FE1 to FE3 and FE2 to FE3 (all *p* < 0.0001, Fig. 1H). Furthermore, standardized FE1 → FE3 effect sizes were large for both multiple-context conditions but small/non-significant for SCFE (Fig. 1I). Together, these findings demonstrate that contextual novelty is not required for the enhanced extinction produced by MCFE. Instead, varying the extinction context is sufficient to achieve the behavioral advantage of multiple-context training.

### Extinction training in multiple contexts limits context-dependent relapse

Because extinction does not erase the original CS–US memory, animals remain susceptible to spontaneous recovery, renewal, and reinstatement [6]. Therefore, we asked whether the behavioral advantage of MCFE extends beyond extinction training to reduce susceptibility to fear relapse. To assess time- and context-dependent relapse, separate cohorts of S1 mice were tested 2 and 30 days after SCFE or MCFE training in either the original FC context, novel contexts, or the familiar extinction context (Fig. 2A).

Fear acquisition did not differ between SCFE and MCFE groups in any cohort (Supplementary Fig. S3), and the enhanced between-session extinction produced by MCFE was replicated across all experiments (Fig. 2A). When mice were tested in the FC context (A), MCFE did not alter freezing behavior at either timepoint (Fig. 2B, left). In contrast, when mice were tested in two novel contexts (E and F), MCFE mice exhibited significantly lower freezing than SCFE mice at the 2-d recall test (*t*[84] = 3.94, *p* < 0.0001), but not the 30-d (*t*[101] = −0.86, *p* = 0.389). Interestingly, SCFE mice exhibited decreased freezing between the 2- and 30-d timepoints (*t*[382] = 4.18, *p* < 0.0001), consistent with additional extinction, whereas MCFE mice increased freezing (*t*[379] = −4.05, *p* < 0.0001), consistent with spontaneous recovery (Fig. 2B, middle). The apparent improvements in SCFE mice between timepoints could reflect either the effect of additional extinction acquired during testing in two novel contexts (i.e., a pseudo-MCFE effect) and/or time-dependent generalization of the extinction context. To distinguish these possibilities, a separate cohort of mice was first tested in the familiar context B (2-d), followed by a novel context F (30-d). Under these conditions, MCFE mice exhibited lower freezing than SCFE mice across testing days (F[1,34] = 7.85, *p* = 0.0083) and no spontaneous recovery was observed, supporting the conclusion that repeated novel-context testing facilitated the convergence seen in the previous cohort. Together, these findings indicate MCFE promotes more generalized extinction recall while attenuating context-dependent fear renewal (Fig. 2B, right).

We next asked whether MCFE protects against fear reinstatement following unsignaled US exposure. We first established that 3 footshocks–but not 1–were sufficient to reinstate freezing (*t*[25] = −3.86, *p* = 0.0021, Fig. 2D, Supplementary Fig. S4A–C). Using this reinstatement protocol, we found that SCFE and MCFE mice exhibited comparable freezing during CS re-exposure (*p* > 0.05, Fig. 2E, Supplementary Fig. S4D–F).

In summary, these findings demonstrate that MCFE selectively enhances the generalization of extinction memory across contexts but does not eliminate all forms of fear relapse. While MCFE protects against context-dependent renewal, it does not prevent relapse driven by US-induced reinstatement or spontaneous recovery.

### MCFE shifts neuronal recruitment toward extinction-related activity while reorganizing reactivated fear networks

To identify neural mechanisms that distinguish multiple-context from single-context extinction, we used a dual Fos-mapping strategy to compare neuronal recruitment during MCFE and SCFE. TRAP2xAi14 mice underwent SCFE or MCFE as in Fig. 1A, with two additional groups: single-session FE (SSFE), in which mice received CS exposure only on FE3 to index neural recruitment during early FE acquisition, and tone exposure without shock (US-) to index background Fos. For both of these control groups, context exposure was counterbalanced, with half of the mice exposed to the same (B;B;B) and half to different (B;C;D) contexts across days. FC-related neural ensembles were tagged using TRAP2, extinction-related activation was quantified with Fos-immunofluorescence after FE, while dual-labeled cells represented FC-tagged neurons reactivated during FE (Fig. 3A–C).

Behavior during the mapping experiment replicated our earlier findings (Figs. 1 and 2): MCFE mice exhibited lower freezing than SCFE on FE3 (*t*[200] = 2.56, *p* = 0.0113), whereas SSFE mice displayed significantly higher FE3 freezing than both SCFE and MCFE. The US- group exhibited minimal freezing throughout testing (Fig. 3B, Supplementary Fig. S5).

Contrary to our hypothesis that MCFE would limit reactivation of FC-tagged cells on FE3, no regional differences in TRAP+, Fos +, or dual+ density were detected between FE groups (Supplementary Fig. S6A, B). The lack of significant US+ versus US- differences despite broad numerical increases in TRAP+ density suggests limited sensitivity of regional density measures, while the 10-d interval between FC tagging and tissue collection may also have permitted fear-ensemble reorganization [33]. Nonetheless, we next explored patterns of neuronal recruitment and network organization, as previous studies have found that these may reflect behavioral differences [43–46].

We compared the relative recruitment of conditioning- and extinction-related neuronal populations. Recruitment bias, defined as fold-change differences between background-normalized Fos+ and TRAP+ densities, qualitatively shifted toward extinction in MCFE, relative to SCFE and SSFE (Fig. 3D, Supplementary Fig. S6D).

We then examined inter-regional co-activation using Spearman correlations (ρ) of TRAP+, Fos+ and dual-labeled densities and visualized MCFE–SCFE difference networks (Δρ = ρMCFE − ρSCFE, Fig. 3E, F, Supplementary Fig. S6E, Supplementary Table 2). Consistent with identical treatment at the time of TRAP labeling, the FC-related network showed less pronounced MCFE-SCFE Δρ than the Fos+ and dual+ labeled networks (Supplementary Fig. S6E). The extinction-related (Fos+) co-activation network was predominantly SCFE-biased, with prefrontal regions emerging as principal differential nodes, including PL (degree = 9, ΣΔρ = −6.58) and ACA (degree = 7, ΣΔρ = −5.90, Fig. 3E). In contrast, the analysis of reactivated FC ensembles (dual-labeled neurons) revealed an MCFE-biased network centered on regions important for contextual processing [7]. Specifically, CA1 emerged as a high-strength MCFE-biased differential node, with high degree (11) and positive-signed node strength (ΣΔρ = 6.98, Fig. 3F). Bootstrap and permutation analyses identified limited above-chance co-activation for extinction-related activity (Fig. 3G). Analyses of FC-reactivation, however, showed broad hippocampal coupling with entorhinal, amygdalar, midbrain, and thalamic regions, as well as prefrontal-midbrain coupling in MCFE (all qFDR ≤ 0.003), whereas SCFE engaged prefrontal-amygdala and prefrontal-thalamic interactions (qFDR ≤ 0.01, Fig. 3H).

Together, these exploratory findings point to hippocampal involvement in MCFE, suggesting that MCFE may alter both extinction-related neural recruitment and organization within the reactivated FC ensembles.

### Chemogenetic inhibition of the dorsal, but not ventral, hippocampus selectively impairs extinction in multiple contexts

The prominent recruitment of hippocampal functional circuits by MCFE prompted us to test whether hippocampal activity is required for enhanced extinction. We compared the contributions of the dorsal hippocampus (dHP), which is rich in place cells and strongly implicated in contextual processing, and the ventral hippocampus (vHP), which contains cells responsive to stimulus identity and intensity [47, 48]. To inhibit hippocampal neurons, we expressed an inhibitory chemogenetic receptor (hM4Di) or a control reporter (mCherry) in either the dorsal (Fig. 4A–C) or ventral hippocampus (Fig. 4D–F).

B6 mice underwent FC and FE as described in Fig. 1A, and animals received C21 (1 mg/kg, *i.p*.) 30 min before FE2, corresponding to the first contextual transition (B → C) in MCFE. FC did not differ between groups (Supplementary Fig. S8).

Chemogenetic inhibition of the dHP selectively disrupted extinction learning in MCFE. HM4Di-expressing MCFE mice exhibited greater freezing than mCherry-expressing controls during both FE2 (*t*[36] = −2.84, *p* = 0.00735) and FE3 (*t*[36] = −3.84, *p* < 0.0001), abolishing the enhanced between-session extinction normally produced by MCFE (Fig. 4B, C). In contrast, dHP inhibition had no effect on SCFE.

Inhibiting the vHP produced a markedly different outcome. vHP inhibition did not impair extinction during MCFE, indicating that vHP activity is not required for the behavioral advantage of MCFE. Although hM4Di-expressing SCFE mice exhibited higher overall freezing than controls (F[1,18] = 5.08, *p* = 0.0368), suggesting possible anxiogenic effects of general vHP inhibition, between-session extinction remained intact (Fig. 4E, F).

Overall, these findings demonstrate that dorsal, but not ventral, hippocampal activity is necessary for the enhanced extinction learning produced by MCFE, providing evidence for a causal role of the dHP in multiple-context extinction learning.

### Dorsal hippocampal dopamine D2-like receptor signaling is required for enhanced extinction during multiple context training

To identify the mechanisms through which the dHP promotes enhanced extinction learning during MCFE, we next examined the contribution of dopamine receptor signaling. Dopaminergic signaling has emerged as an important regulator of extinction learning, prediction error, and memory updating [29–31, 49–51]. Because the dHP supports contextual encoding and was required during the first contextual shift in extinction, we hypothesized that this shift transiently increases expectation of the US, allowing its omission to engage dopamine-dependent updating of the extinction memory.

To test this, hybrid S1/B6 mice underwent FC and FE as in Fig. 1A, except that D1- or D2-like receptor (D1R or D2R) antagonists were administered before FE2 (Fig. 5A), either systemically (Fig. 5B, C) or locally into dHP (Fig. 5D–F). All groups exhibited comparable fear acquisition and fear recall before extinction, and dopamine antagonism did not affect locomotion (Supplementary Fig. S9).

Systemic D1-like receptor blockade prevented between-session extinction in SCFE mice (*t*[451] = 0.25, *p* = 0.802), whereas vehicle- and D2R antagonist-treated SCFE mice showed normal extinction. In contrast, all MCFE groups exhibited robust FE1 → FE3 reductions regardless of treatment (Fig. 5B, C). These findings support distinct roles for dopamine signaling in SCFE and MCFE; while systemic D1-like receptor-mediated signaling appears to be necessary for extinction under SCFE, MCFE was resistant to any disruption in learning by systemic D1R blockade.

In contrast, local D2-like receptor blockade within the dHP selectively impaired extinction learning in the MCFE condition. D2-like antagonist-treated MCFE mice failed to reduce freezing across days (*t*[373] = 1.76, *p* = 0.158) and exhibited higher freezing than vehicle and D1-like antagonist-treated mice on FE3 (both *p* < 0.01, Fig. 5E, F). Neither antagonist altered extinction during SCFE.

Considering its key role in extinction, we additionally manipulated dopamine receptor activation in the medial prefrontal cortex (mPFC), with infusions targeted to the ILA [52]. Local infusion of D1-like or D2-like receptor antagonists on FE2 into the mPFC had no detectable effects on extinction learning under either SCFE or MCFE (Fig. 5G–I).

Taken together, these findings demonstrate that dopamine D2-like receptor-signaling within the dHP is specifically required for the enhanced extinction produced by MCFE.

## DISCUSSION

A major limitation of extinction-based therapies is the vulnerability of safety learning to relapse, particularly following changes in context. Here, we established an MCFE paradigm that strengthens extinction learning across training and produces a more generalizable extinction memory at recent and remote timepoints in two mouse strains that differ in stress-susceptibility (129S1 and C57BL/6J, Figs. 1 and 2). Although contextual novelty has been shown to improve learning and memory, including extinction recall, it was not necessary for MCFE to enhance extinction (Fig. 1) [53–56]. The enhancement produced by MCFE was nevertheless selective: it reduced context-dependent fear renewal but did not prevent US-induced reinstatement, suggesting that contextual variation promotes extinction generalization rather than broad resistance to relapse. We hypothesize that MCFE may broaden contextual associations with the CS-no-US contingency and promote memory updating when the CS is encountered in unexpected contextual settings.

Fos-mapping revealed reorganization of FC ensembles reactivated during MCFE centered on hippocampal networks (Fig. 3). Consistent with this network-level pattern, chemogenetic inhibition of the dHP eliminated the MCFE enhancement while leaving SCFE unaffected, establishing a causal role for this region in mediating MCFE (Fig. 4).

These findings extend evidence that the dHP supports contextual encoding of fear extinction [57–62]. Previous work has shown that dHP inactivation during extinction attenuated acquisition and produced later renewal even in the extinction context, suggesting impaired binding of the CS-no-US association to its training context [63]. Our findings suggest that this hippocampal contribution becomes especially important when the CS-context relationship changes: dHP inhibition during the FE2 contextual shift attenuated extinction and eliminated the MCFE enhancement, whereas SCFE remained unaffected. This selectivity is consistent with the proposal that the hippocampus is particularly engaged by associative novelty—the occurrence of a familiar CS in a context in which it is not expected [28]. Although vHP inhibition increased overall freezing in SCFE, it did not impair MCFE, suggesting a contribution to fear expression distinct from dHP-dependent contextual updating [47, 64, 65].

Dopaminergic signaling may provide a mechanism through which exposure to the CS in an unexpected context promotes updating. D2-like receptor blockade in the dHP abolished the MCFE enhancement (Fig. 5). Because dHP predominantly expresses D2, but not D3, receptors, these findings implicate D2R-signaling in this process [66]. Critically, D2R blockade during the FE2 contextual shift did not affect extinction during FE2 but it did attenuate extinction on FE3, whereas dHP inhibition impaired extinction on both days (Figs. 4 and 5). Thus, dHP activity appears to support behavioral adaptation during a contextual transition as well as subsequent memory updating, whereas D2R-signaling more specifically supports updating. Together with evidence that contextual manipulations can increase hippocampal dopamine and facilitate extinction, these findings support a model in which associative novelty recruits hippocampal dopamine to update extinction across contexts [56]. Future studies should define how dHP D2Rs and dopaminergic inputs from the ventral tegmental area and locus coeruleus contribute to this process [66–70].

Interestingly, the dopaminergic mechanisms supporting MCFE appear to differ from those underlying SCFE. Whereas dHP D2R signaling was necessary for MCFE, systemic D1-like receptor blockade selectively disrupted SCFE (Fig. 5). This effect was not reproduced by local D1-like receptor blockade in either dHP or mPFC, leaving its neural locus unresolved. Interpretation of the mPFC manipulation was further limited by PL damage and variability in infusion placement relative to ILA.

Together, these findings suggest that MCFE reduces the context specificity of extinction by promoting memory updating, providing mechanistic support for emerging behavioral strategies aimed at improving the generalizability of exposure-based therapies.

## Supplementary Material

Supplemental

**Supplementary information** The online version contains supplementary material available at https://doi.org/10.1038/s41386-026-02577-x.

## Figures and Tables

**Fig. 1:**
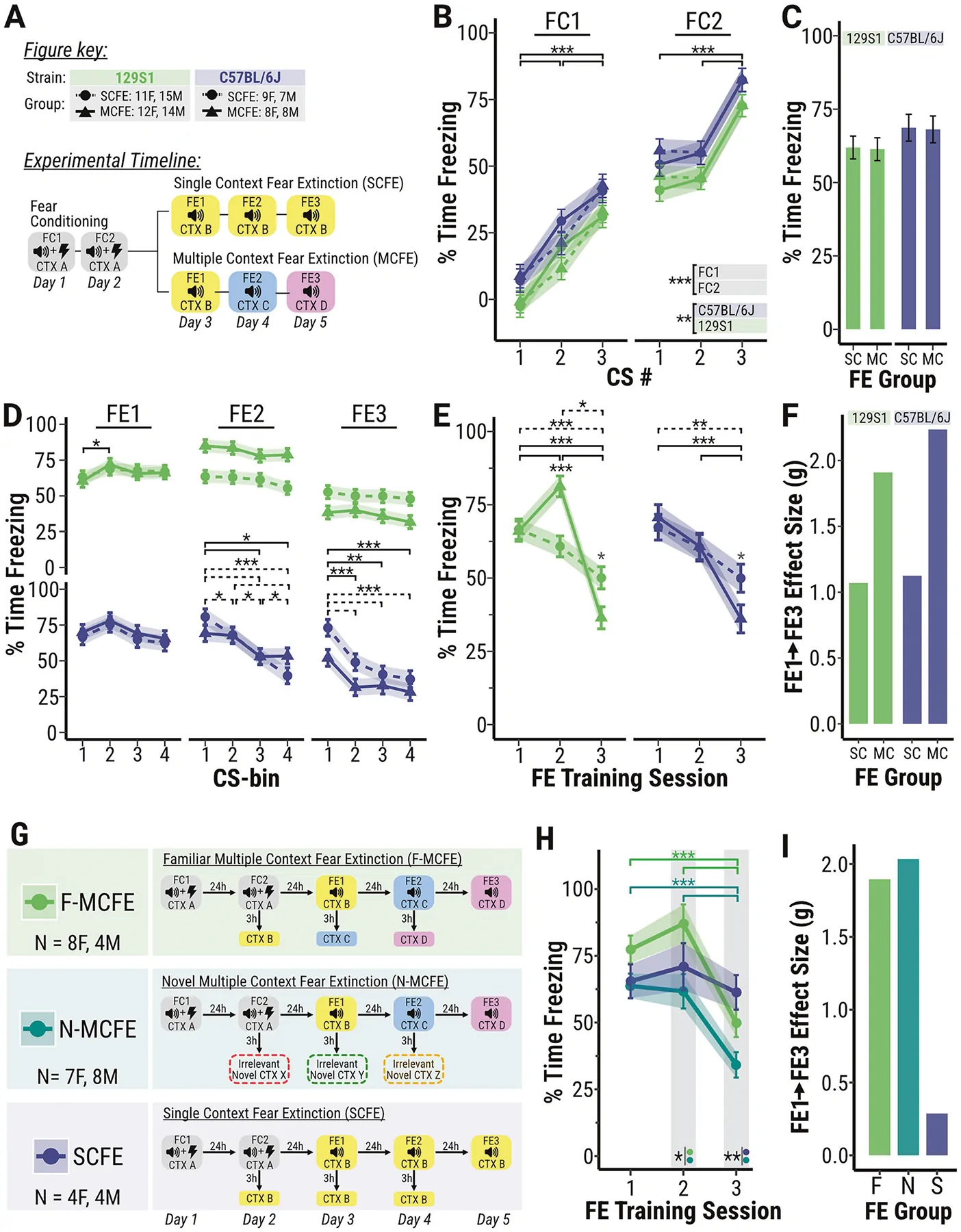
Training in multiple contexts enhances extinction learning without requiring contextual novelty. **A** Experimental timeline and figure key. **B** Cued fear conditioning (FC). Three CS-US pairings on consecutive training days (FC1, FC2) increased conditioned freezing in both C57BL/6J (B6) and 129S1 (S1) mice from CS1 to CS3 (all *p* < 0.0001) and from FC1 to FC2 (F[1,410] = 307.26, *p* < 0.0001). **C** Fear recall at the start of extinction (FE1). Freezing to the first 3 CS did not differ between groups (F[1,80] = 0.01, *p* = 0.906), confirming comparable conditioned fear before extinction. **D**, **E** Extinction sessions (FE1–FE3) were conducted in either a single context (SCFE) or multiple contexts (MCFE). **D** Within-session extinction. Freezing was averaged in three-CS bins and compared within each session. A strain x bin interaction was observed (F[3,720] = 16.21, *p* < 0.0001), wherein only B6 mice showed significant within-session decreases on FE2 and FE3, independent of extinction type. **E** Between-session extinction. All groups exhibited significant reductions in freezing across extinction days (FE1 → FE3, all *p* < 0.05). MCFE mice exhibited lower levels of freezing on FE3 (both *p* < 0.05), with S1 MCFE animals showing a transient increase in freezing on FE2 (context shift B → C, *t*[80] = −3.42, *p* = 0.000973), consistent with renewal, followed by a sharp decline on FE3. **F** Standardized effect size (g) for the FE1 → FE3 reduction in freezing. MCFE mice showed larger across-session decreases (S1: *g* = 1.91, *p* < 0.0001; B6: *g* = 2.24, *p* < 0.0001) than SCFE mice (S1: *g* = 1.07, *p* = 0.000291; B6: *g* = 1.12, *p* = 0.00331). **G** Experimental timeline and figure key for novelty experiment. Procedures matched those in this Figure, except for the pre-exposure 24 h before extinction sessions: F-MCFE mice were pre-exposed to the subsequent extinction contexts, N-MCFE mice to an irrelevant context (leaving extinction context novel), and SCFE mice to context B. S1 mice used for these experiments. **H** Between-session extinction. Both N-MCFE and F-MCFE reduced freezing from FE1 → FE3 (N-MCFE: *t*[38] = 6.55, *p* < 0.0001; F-MCFE: *t*[38] = 5.46, *p* < 0.0001) and FE2 → FE3 (N-MCFE: *t*[38] = 4.88, *p* < 0.0001; F-MCFE: *t*[38] = 5.89, *p* < 0.0001), whereas SCFE showed no across-day reduction (all *p* > 0.05). On FE2, N-MCFE froze less than F-MCFE (*t*[38] = 2.62, *p* = 0.0375), but by FE3, the only difference in freezing was between N-MCFE and SCFE (*t*[38] = −3.38, *p* = 0.00509). **I** Standardized effect size (g) for the FE1 → FE3 reduction. Both MCFE conditions showed larger across-session decreases than SCFE (N-MCFE: *g* = 2.03, *p* < 0.00001; F-MCFE: *g* = 1.90, *p* < 0.0001; SCFE: *g* = 0.29, *p* = 0.925). g = a standardized mean difference, computed as the FE1–FE3 contrast divided by the model residual SD. Shaded areas show EMM ± SEM. Sample sizes (panel (**A**)). Asterisk denotes significant effects (*p < 0.05, **p < 0.01, ***p < 0.001) from a Type III ANOVA with Holm-adjusted contrasts.

**Fig. 2:**
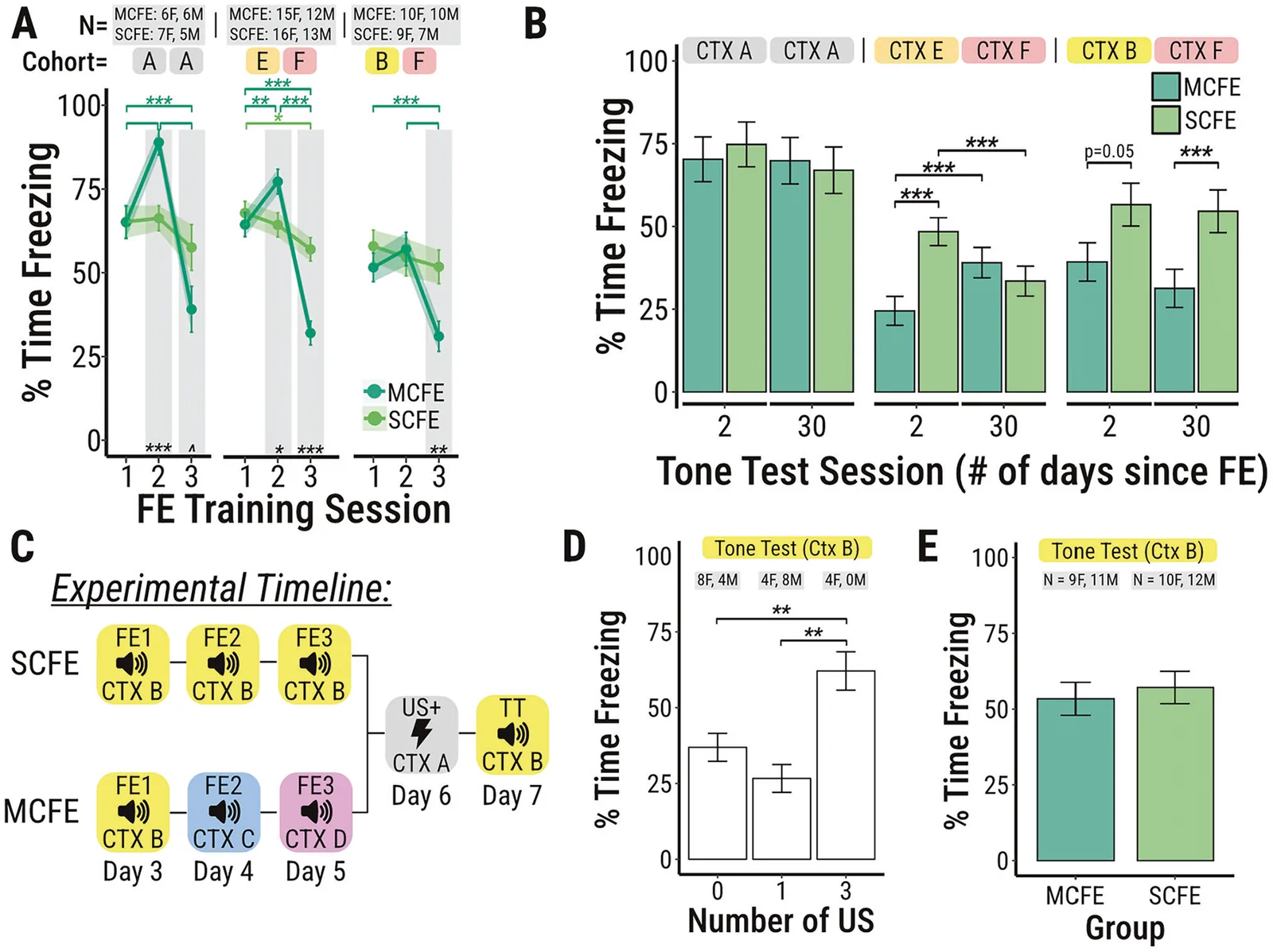
Extinction training in multiple contexts limits the return of fear in novel contexts. **A** Between-session extinction. Procedures matched Fig. 1. As before, groups were treated identically up to extinction, and MCFE mice showed lower freezing than SCFE by FE3 (all *p* < 0.06, see Fig. 1). **B** Fear recall was tested at 2 days (recent) and 30 days (remote) in three cohorts: testing in (**A**) (conditioning context), in novel contexts (**E**, **F**), or extinction context B at 2 d, novel context F at 30 d. Left: No group differences in context A at either timepoint (F[1,28] = 0.01, *p* = 0.925). Middle: In novel contexts, MCFE exhibited lower freezing than SCFE at 2 d (*t*[84] = 3.94, *p* = 0.000168), but the group difference was absent at 30 d (*t*[101] = −0.86, *p* = 0.389); MCFE increased freezing from 2 to 30 d (*t*[379] = −4.05, *p* < 0.0001) while SCFE decreased freezing (*t*[382] = 4.18, *p* < 0.0001). Right (B,F): A group main effect was observed (F[1,34] = 7.85, *p* = 0.00833); MCFE tended to freeze less at 2 d in B (*t*[59] = 2.00, *p* = 0.0502) and showed lower freezing than SCFE at 30 d in F (*t*[59] = 2.69, *p* = 0.00941). **C** Reinstatement timeline. **D** Unsignaled US exposure in context A followed by testing in B: three footshocks**—**but not one**—**reinstated freezing (*t*[25] = −3.86, *p* = 0.0021). **E** After 3-US reinstatement, MCFE and SCFE showed comparable freezing (F[1,80] = 0.39, *p* = 0.537). S1 mice used for these experiments, conventions as in Fig. 1, ^ denotes *p* = 0.06.

**Fig. 3:**
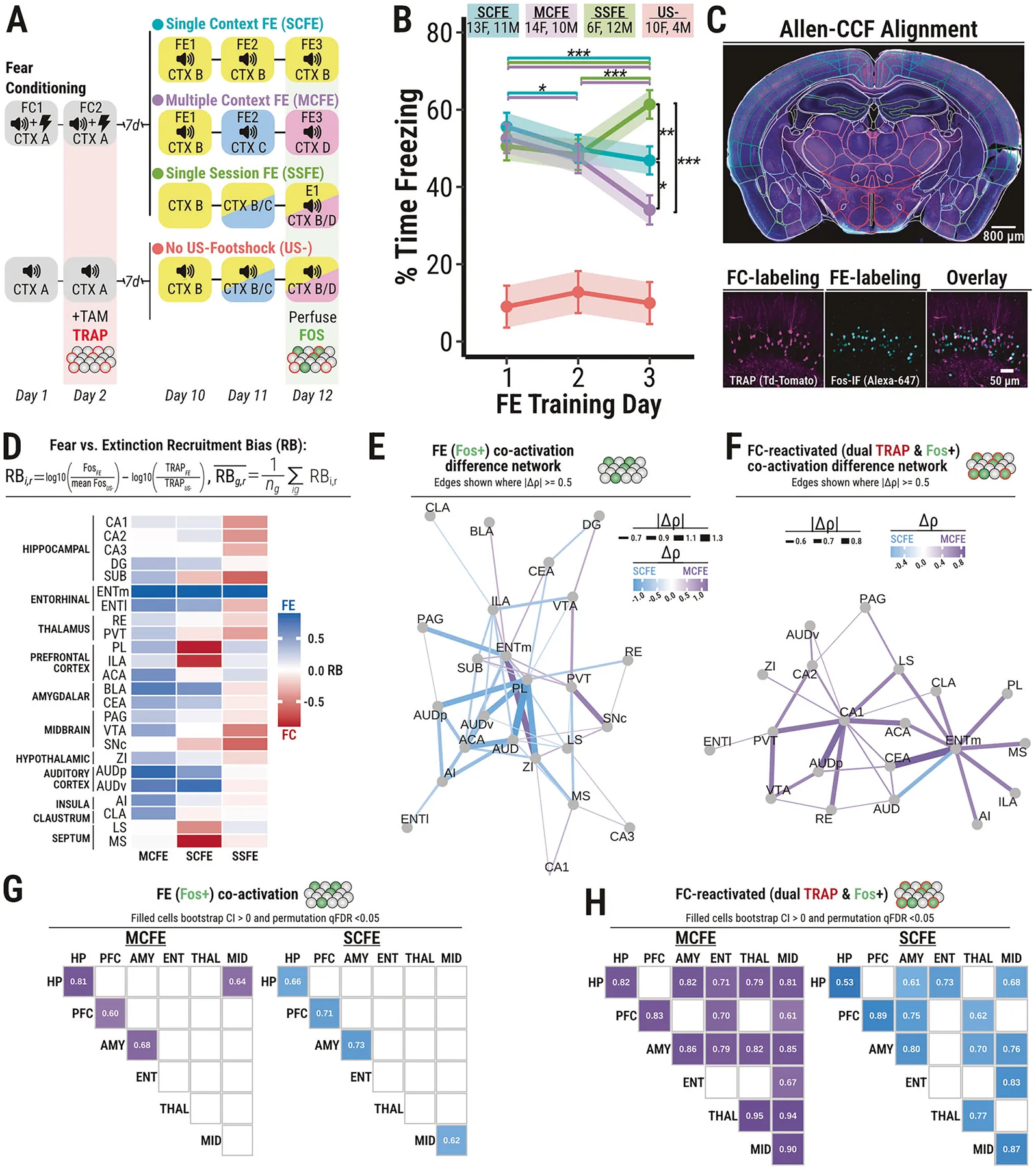
MCFE reorganizes FE (Fos+) and FC-reactivated (Dual+) co-activation networks. **A** Experimental timeline. As in Fig. 1A, with two exceptions. (1) Two additional groups were included: US− (context + tone exposure; no shocks) and single session FE (SSFE), mice in these additional groups were either exposed to the same (B;B;B) or different (B;C;D) contexts each day; (2) all mice received tamoxifen on FC2 to TRAP activity and were perfused 90 min after FE3 for Fos immunolabeling of extinction-related activity. **B** Between session extinction. As before, MCFE produced lower freezing on FE3 compared to SCFE (*t*[200] = 2.56, *p* = 0.0113). The US- group that never underwent FC exhibited significantly lower freezing than all other groups across all days of FE (all *p* < 0.01). SSFE exhibited the highest freezing on FE3, effectively its first day of FE training (SSFE-SCFE: *t*[583] = 3.51, *p* = 0.00129; SSFE-MCFE: *t*[323] = 5.84, *p* < 0.0001). Note, for SSFE on E1–E2, bins correspond to the temporal locations of CS presentations in the other groups, although no CS was delivered. **C** Representative section and Fos-labeling. **D** Heatmap of FC vs. FE neural recruitment bias, defined as the difference between background (US-) normalized regional Fos+ and TRAP+ densities, showing qualitative evidence of preferential FE recruitment in MCFE > SCFE > SSFE. Regional coactivation difference graph for FE (Fos+, **E**) or FC-reactivated (TRAP+ & Fos+, **F**) derived from inter-regional Spearman correlations (ρ) comparing FE groups (Δρ = ρMCFE − ρSCFE, thresholded by |Δρ | > 0.5 for visualization, comparisons with SSFE in Supplementary Fig. S7 and Supplementary Tables 3, 4). Differential co-activation patterns were dominated by SCFE for FE, with PL demonstrating high network centrality (degree = 7, ΣΔρ = −5.90). Differential co-activation patterns for FC-reactivated neurons were dominated by a CA1 MCFE-biased profile (degree = 10, ΣΔρ = 2.40). Parent-system-level bootstrap/permutation analyses of Fos- (**G**) and dual-labeled (**H**) densities supported broad above-chance coordinated reactivation in MCFE for HP and PFC–midbrain coupling, and in SCFE for PFC–amygdala and PFC–thalamus coupling; all qFDR < 0.05 unless noted. B6 mice used for these experiments, conventions as in Fig. 1. Sample sizes for Fos-mapping: MCFE 5 F, 5 M; SCFE 6 F, 4 M; SSFE 1 F, 4 M; US- 5 F, 3 M. Standardized Allen CCF anatomical abbreviations: dentate gyrus (DG), subiculum (SUB), medial entorhinal area (ENTm), lateral entorhinal area (ENTl), nucleus reuniens (RE), paraventricular nucleus of the thalamus (PVT), prelimbic area (PL), infralimbic area (ILA), anterior cingulate area (ACA), basolateral amygdalar nucleus (BLA), central amygdalar nucleus (CEA), periaqueductal gray (PAG), ventral tegmental area (VTA), compact part of substantia nigra (SNc), zona incerta (ZI), primary auditory area (AUDp), ventral auditory area (AUDv), agranular insular area (AI), claustrum (CLA), lateral septal nucleus (LS), medial septal nucleus (MS). Parent anatomical formations grouped as hippocampus (HP), prefrontal cortex (PFC), amygdala (AMY), entorhinal cortex (ENT), thalamus (THAL), and midbrain (MID).

**Fig. 4:**
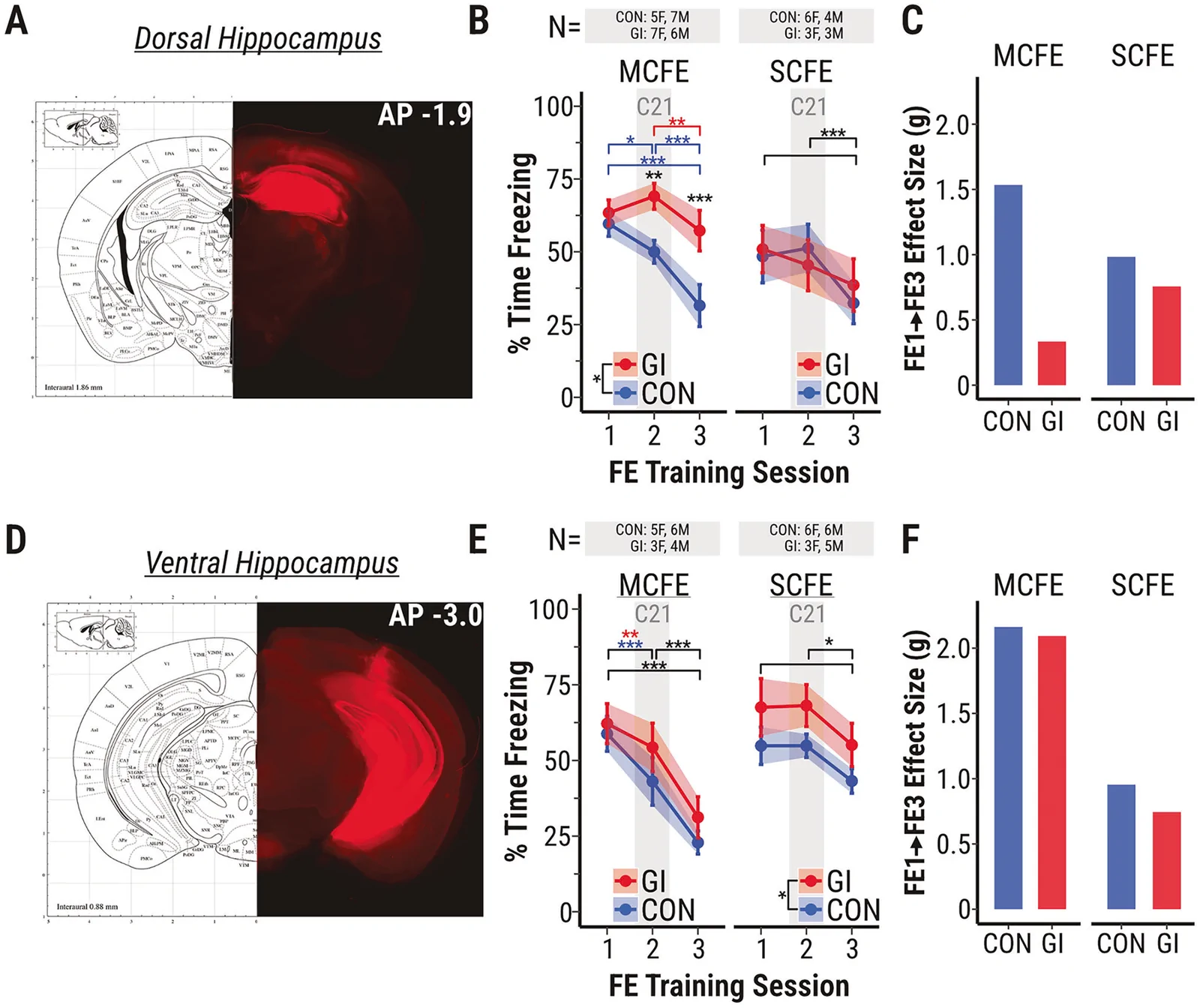
Chemogenetic inhibition of the dorsal but not ventral hippocampus disrupts extinction in multiple contexts. **A** Dorsal hippocampal (dHP) viral transduction. Mice received bilateral injections of AAV5-CaMKII-hM4D(Gi)-IRES-mCitrine (Gi) or AAV5-CaMKII-mCherry (CON). Composite overlays of fluorescence images for all Gi mice. **B** Between-session extinction, dHP. Fear conditioning (data not shown) and fear extinction (FE1–FE3) were as in Fig. 1, except all mice received the DREADD agonist 21 (C21; 1 mg/kg i.p.) 30 min before FE2. In the multiple-context fear-extinction (MCFE) cohort, Gi mice froze more than CON mice on FE2 (*t*[36] = −2.84, *p* = 0.0074) and FE3 (*t*[36] = −3.84, *p* < 0.0001) and failed to reduce freezing from FE1 to FE3 (*t*[273] = 1.71, *p* = 0.178). In the single-context fear-extinction (SCFE) cohort, Gi and CON groups did not differ (F[1,14] = 0.01, *p* = 0.932). **C** Standardized effect size (g) for the FE1 → FE3 reduction in freezing, dHP. Gi MCFE group (*g* = 0.33, *p* = 0.178) had smaller effect size than CON MCFE (*g* = 1.53, *p* < 0.0001). CON MCFE effect size larger than both SCFE groups (Gi SCFE: *g* = 0.76, *p* < 0.0001; CON SCFE: *g* = 0.98, *p* < 0.0001). **D** Ventral hippocampal (vHP) viral transduction. Composite overlays of fluorescence images for all Gi mice. **E** Between-session extinction, vHP. C21 produced no virus-specific effect in MCFE (F[1,16] = 1.77, *p* = 0.202). We observed a main effect of virus conditions in SCFE wherein Gi mice exhibited higher freezing than CON mice (F[18] = 5.08, *p* = 0.0368). **F** Standardized effect size (g), vHP. Both MCFE groups (Gi: *g* = 2.09, *p* < 0.0001; CON: *g* = 2.16, *p* < 0.0001) had larger effect sizes than SCFE groups (Gi: *g* = 0.74, *p* < 0.0001; CON: *g* = 0.95, *p* < 0.0001). B6 mice used for these experiments, conventions as in Fig. 1.

**Fig. 5:**
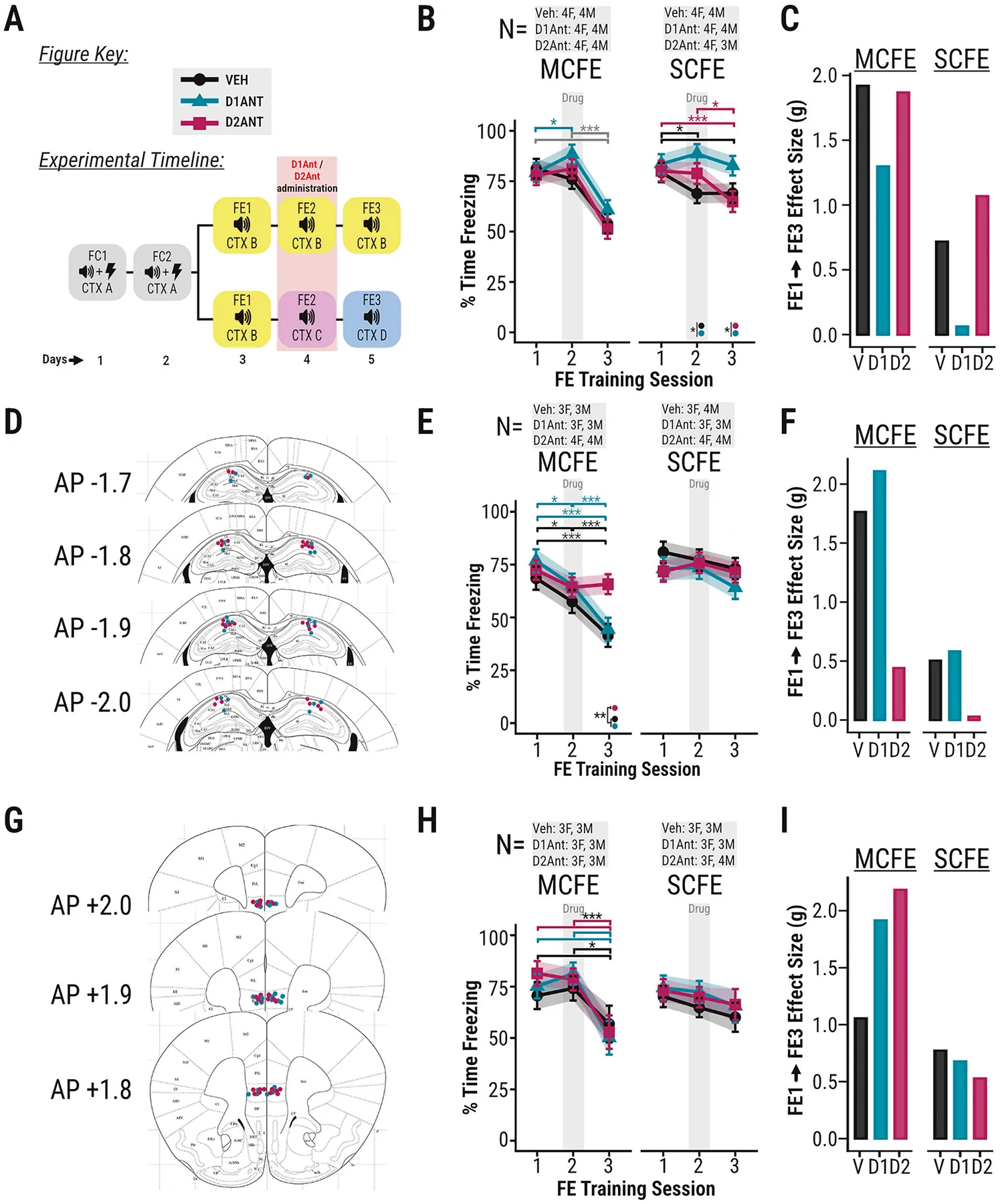
Dopamine D2 receptor blockade in the dorsal hippocampus disrupts extinction in multiple-contexts. **A** Experimental timeline. As in Fig. 1A, except mice received an infusion of a D1-like receptor antagonist (SCH23390), D2-like receptor antagonist (raclopride), or saline either systemically or intracranially into the mPFC or dHP through bilateral cannulae prior to the FE2 behavioral session. **B** Between-session extinction for systemic drug administration. SCFE mice receiving the D1-like receptor antagonist failed to reduce freezing from FE1 to FE3 (*t*[451] = 0.25, *p* = 0.802) and exhibited higher freezing than vehicle SCFE mice on FE2 (*t*[59] = −2.88, *p* = 0.0163) and than D2R SCFE mice on FE3 (*t*[60] = 2.53, *p* = 0.0422). **C** Standardized effect size (g) for the FE1 → FE3 reduction in freezing with systemic drug administration. All MCFE groups had larger effect sizes (Veh: *g* = 1.92, *p* < 0.0001; D1Ant: *g* = 1.30, *p* = 0.012; D2Ant: *g* = 1.87, *p* < 0.0001) than SCFE groups (Veh: *g* = 1.21, *p* = 0.011; D1Ant: *g* = 0.06, *p* = 0.802; D2Ant: *g* = 1.07, *p* = 0.00025), with the D1Ant SCFE group having approximately no reduction in freezing across days. **D** dHP infusion sites for D1-like and D2-like receptor antagonist infusions. **E** Between-session extinction for intra-dHP administration. MCFE mice receiving the D2R antagonist failed to reduce freezing from FE1 to FE3 (*t*[373] = 1.76, *p* = 0.158) and exhibited higher freezing than D1Ant and vehicle MCFE groups on FE3 (Veh-D2Ant: *t*[55] = −3.38, *p* = 0.00399; Veh-D1Ant: *t*[55] = −2.97, *p* = 0.00874). No effect of drug administration on SCFE (all *p* > 0.05). **F** Standardized effect size (g), dHP. D2R MCFE group had a much smaller effect size (*g* = 0.44, *p* = 0.158) than the vehicle (*g* = 1.76, *p* < 0.0001) and D1Ant MCFE (*g* = 2.11, *p* < 0.0001) groups. **G** mPFC infusion sites for D1-like and D2-like receptor antagonist infusions. **H** Between-session extinction for intra-mPFC administration. All MCFE groups decreased freezing from FE1 to FE3 (Veh: *t*[39] = 2.53, *p* = 0.0466; D1Ant: *t*[33] = 4.97, *p* < 0.0001; D2Ant: *t*[32] = 5.73, *p* < 0.0001), while SCFE groups showed no reductions in freezing across days (all *p* > 0.05). No effect of dopamine antagonism (F[2,31] = 0.33, *p* = 0.72). **I** Standardized effect size (g), mPFC. All MCFE groups had larger effect sizes than their SCFE counterparts (Veh: *g* = 1.06 vs. 0.77; D1Ant: *g* = 1.91 vs. 0.68; D2Ant: *g* = 2.18 vs. 0.53). Hybrid S1/B6 mice used for these experiments, conventions as in Fig. 1.

## Data Availability

A previous version of this article was published as a preprint on bioRxiv: https://www.biorxiv.org/content/10.64898/2026.01.18.700199v1. Data and analysis scripts are available at: https://doi.org/10.6084/m9.figshare.33000362.
