# Supplementary material for: Multiple-context extinction strengthens fear suppression through hippocampal dopamine D2R-dependent memory updating
Source: Neuropsychopharmacology. Author manuscript; Available in PMC 2026 Oct 22. (PMC13689360; doi:10.1038/s41386-026-02577-x)
Supplement: Supplementary file 1 [file NIHMS2216335-supplement-Supplemental.pdf]

Supplemental Figures

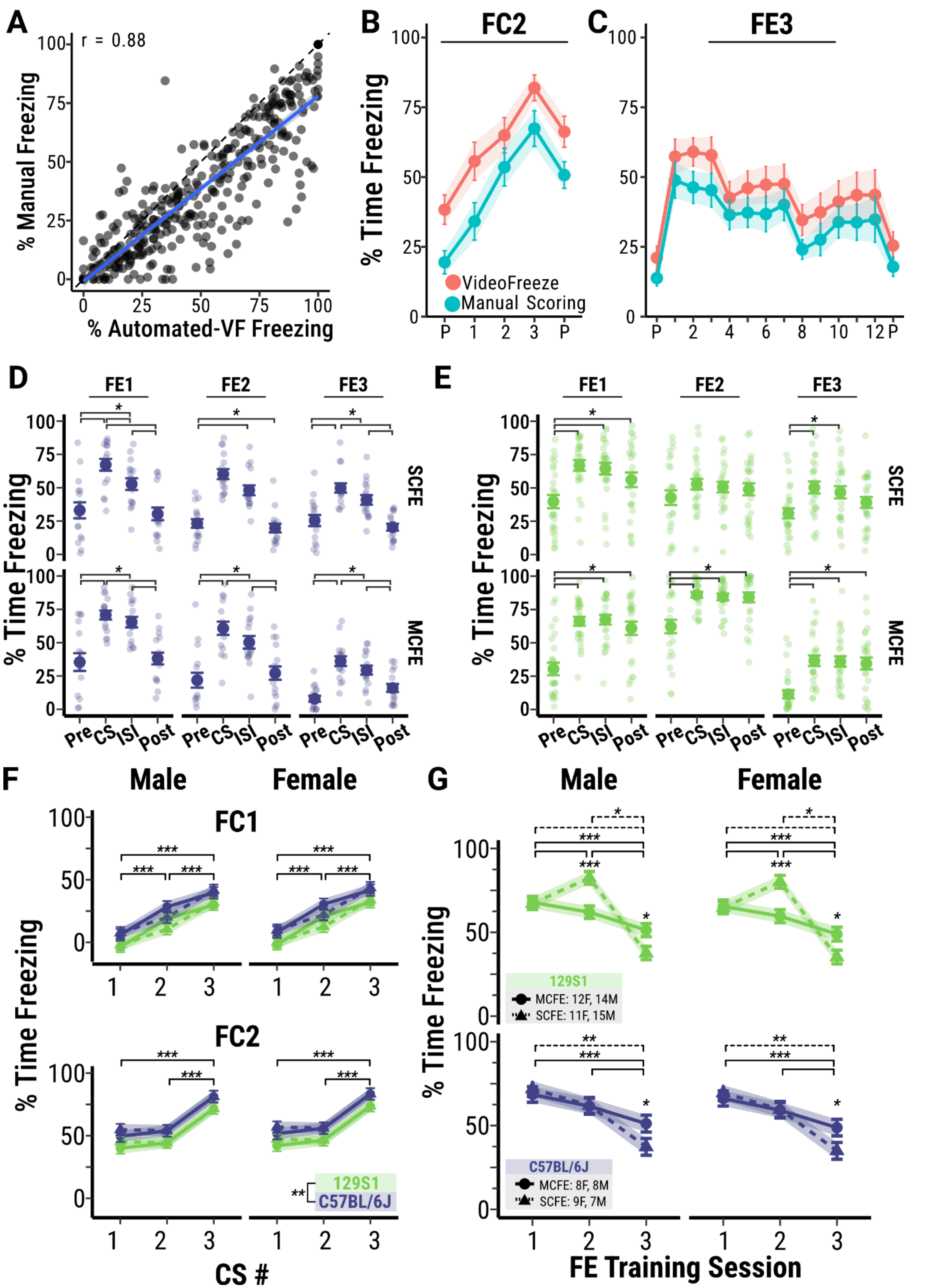
A
r = 0.88
% Manual Freezing
% Automated-VF Freezing
B
FC2
C
FE3
% Time Freezing
VideoFreeze
Manual Scoring
D
FE1
FE2
FE3
SCFE
MCFE
Pre
CS
ISI
Post
E
F
Male
Female
FC1
FC2
129S1
C57BL/6J
CS #
G
MCFE: 12F, 14M
SCFE: 11F, 15M
MCFE: 8F, 8M
SCFE: 9F, 7M
FE Training Session

Supplemental Figure 1. Validation of fear-related measures.
(A–C) Comparison of automated *VideoFreeze* and manual freezing quantification using BORIS. Percent time freezing quantified by each method is shown across trial periods during 20 FC2 trials **(B)** and 20 FE3 trials **(C)**. *VideoFreeze* and manual freezing estimates were strongly correlated across observations (Pearson's r=0.88; n= 20 animals, 40 videos, **A**), with similarly strong correlations when analyzed by trial type (FC2: r=0.85; E3: r=0.89) or extinction group (MCFE: r=0.89; SCFE: r=0.87). **(D–E)** Comparison of freezing across Pre-CS (Pre), CS, brief interstimulus intervals (ISI, 5-22 s), and Post-CS (Post) periods during FE training for B6 **(D)** and S1 **(E)** mice. Across groups, mice exhibited greater freezing during the CS than during the Pre period. B6 mice also exhibited greater freezing during the CS than during the ISI, particularly early in extinction training. In contrast, S1 mice showed sustained freezing across the CS, ISI, and Post periods. **(F–G)** Behavior stratified by sex. **(F)** Cued fear conditioning (FC). While B6 animals exhibited higher freezing across FC trials ($F[1,80]=11.25$, p=0.00122), all animals increased freezing across the three CS–US pairings across both days. No effect of sex ($F[1,80]=0.55$, p=0.462) or extinction group ($F[1,80]=0$, p=0.967) were observed. **(G)** Between-session extinction. All animals successfully decreased freezing from FE1 to FE3, with MCFE animals exhibiting lower freezing on FE3 and MCFE S1 mice exhibiting a transient increase in freezing from FE1 to FE2. There was no effect of sex on these behavior patterns ($F[1,79]=0.72$, p=0.4). All future analyses did not stratify by sex in the linear mixed-effects model. Conventions as in Fig. 1. Supplement to **Fig. 1**.

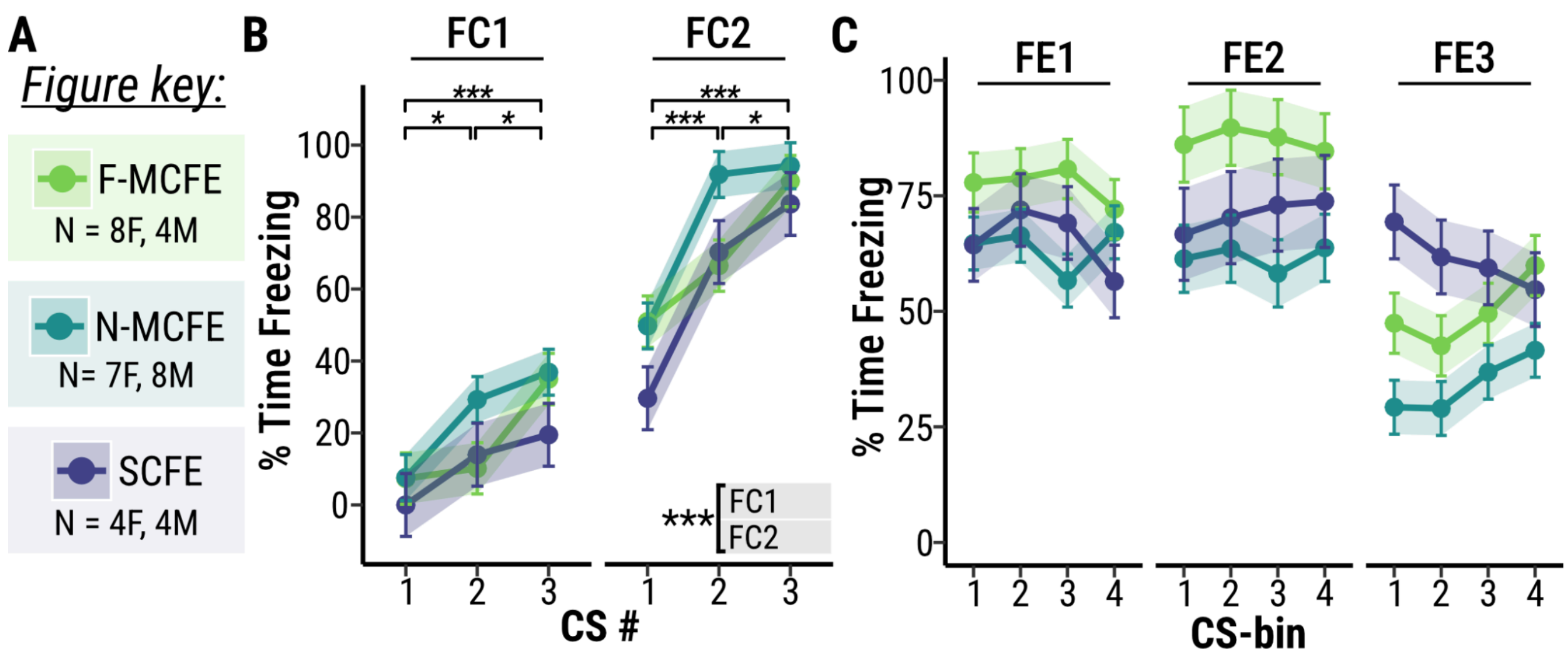

Supplemental Figure 2. FC and FE behavior for novelty experiments.
(A) Figure key. **(B)** Fear conditioning (FC). CS-US pairings on consecutive training days (FC1, FC2) increased conditioned freezing across CS presentations ($F[2,160]=46.39$, $p<0.0001$) and FC days ($F[1,160]=287.39$, $p<0.0001$) irrespective of extinction group ($F[2,32]=2.89$, $p=0.0705$). **(C)** Within-session extinction. Freezing (averaged in three-CS bins) showed no significant within-session decreases ($F[3,315]=0.06$, $p=0.98$). S1 mice used for these experiments, conventions as in Fig. 1; g and SEM as defined there. Supplement to **Fig. 1**.

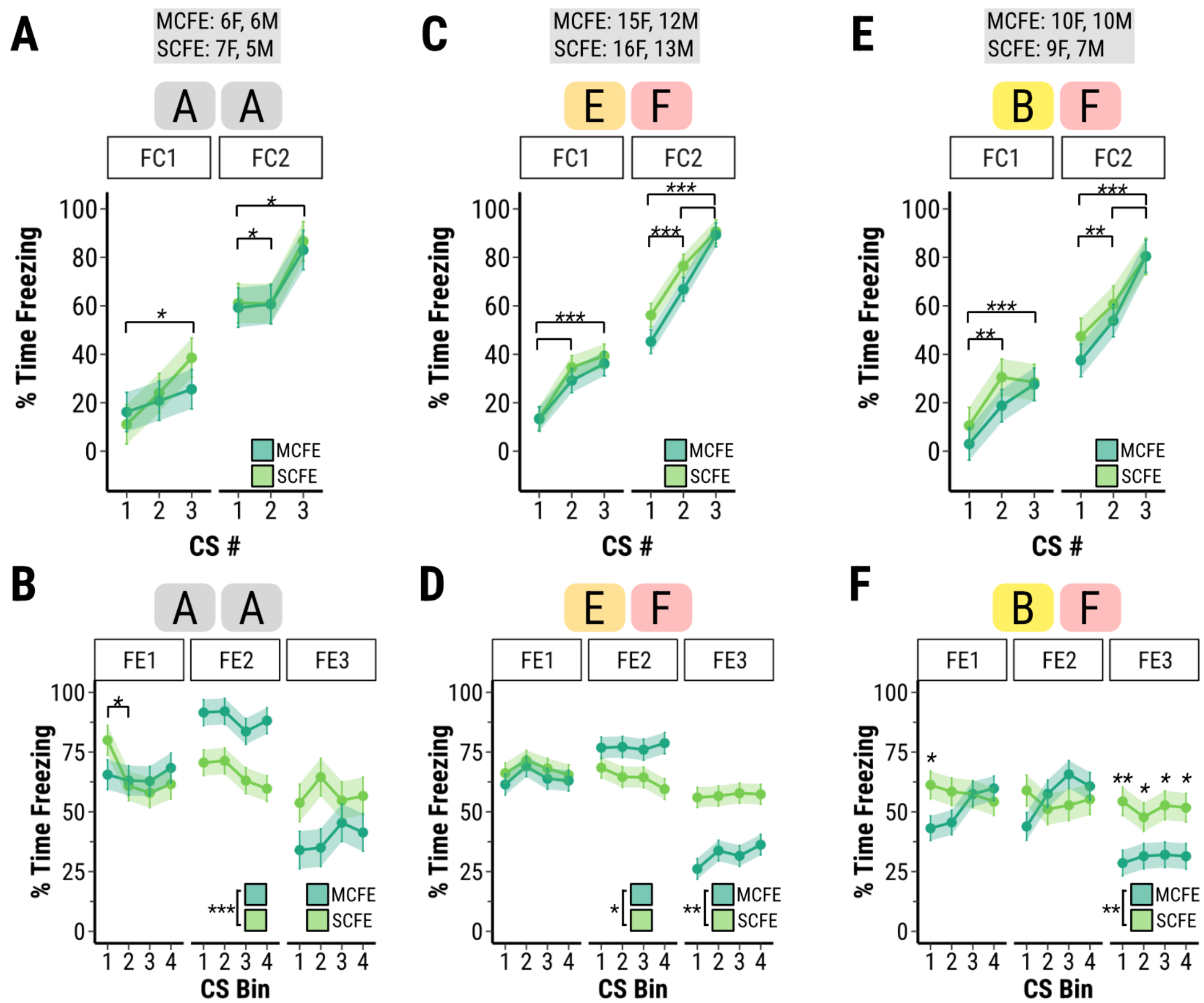

Supplemental Figure 3. FC and FE behavior of mice tested in distinct recall contexts. (A, C, E) Cued fear conditioning (FC). All mice increased freezing from FC1 to FC2, regardless of extinction group (SCFE, MCFE) or recall test cohort AA (F[1,130]=148.97, *p*<0.0001, **A**), EF (F[1,301]=290.19, *p*<0.0001 **C**), or BF (F[1,170]=187.26, *p*<0.0001, **E**). **(B, D, F)** Within-session extinction for the AA cohort **(B)**, EF cohort **(D)**, and BF cohort **(F)**. **(B)** In the AA cohort, all MCFE mice showed a significant reduction in freezing from FE1 to FE3 (*t*[30]=4.92, *p*<0.0001) and FE2 to FE3 (*t*[30]=8.95, *p*<0.0001), with a transient increase in freezing from FE1 to FE2 (*t*[30]=-6.34, *p*<0.0001). SCFE mice showed no changes in freezing across days. **(D)** In the EF cohort, both MCFE and SCFE mice showed a decrease in freezing across days, with SCFE mice decreasing from FE1 to FE3 (*t*[66]=3.06, *p*=0.00954) and MCFE mice decreasing from FE1 to FE3 (*t*[66]=8.86, *p*<0.0001) and FE2 to FE3 (*t*[66]=11.18, *p*<0.0001), with a transient increase in freezing from FE1 to FE2 (*t*[66]=-3.29, *p*=0.0016). **(F)** In the BF cohort, MCFE mice decreased freezing from FE1 to FE3 (*t*[38]=5.06, *p*<0.0001) and FE2 to FE3 (*t*[38]=6.09, *p*<0.0001), whereas SCFE mice showed no differences in freezing across days. S1 mice used for this experiment, conventions as in Fig. 1. Supplement to **Fig. 2**.

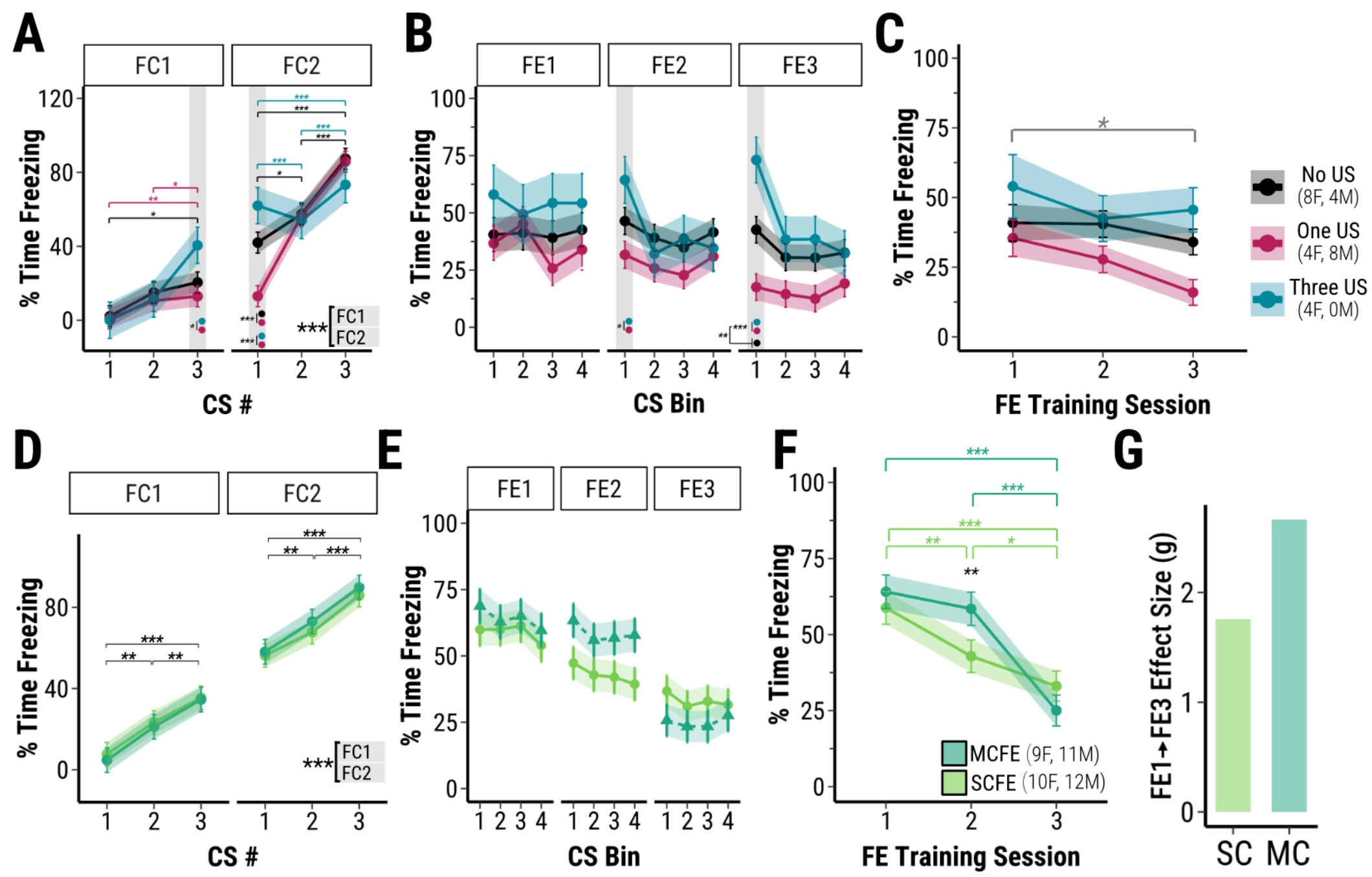

Supplemental Figure 4. FC and FE behavior during evaluation of different reinstatement protocols (0, 1, 3 unsignaled US) and effect of 3US protocol on fear recall.
(A) Cued fear conditioning (FC) for reinstatement protocol evaluation. All mice increased freezing from FC1 to FC2, regardless of reinstatement group ($F[1,125] = 215$, $p<0.0001$). **(B)** Within-session extinction. **(C)** Between-session extinction. All mice decreased freezing from FE1 to FE3, regardless of reinstatement group ($t[31]=3.04$, $p=0.0142$). **(D)** FC for reinstatement test. All mice increased freezing from FC1 to FC2 ($F[1,202]=345.64$, $p<0.0001$), with no effect of extinction group ($F[1,84]=0.02$, $p=0.875$). **(E)** Within-session extinction. **(F)** Between-session extinction. Both SCFE and MCFE mice decreased freezing across days, with SCFE mice decreasing freezing from FE1 to FE2 ($t[54]=3.51$, $p=0.00183$), FE2 to FE3 ($t[52]=2.12$, $p=0.0385$), and FE1 to FE3 ($t[54]=5.05$, $p<0.0001$), and MCFE mice only decreasing freezing from FE1 to FE3 ($t[55]=7.41$, $p<0.0001$) and FE2 to FE3 ($t[52]=7.01$, $p<0.0001$), consistent with an attenuated renewal effect. **(G)** Standardized effect size (g) for the FE1→FE3 reduction in freezing. MCFE mice showed larger across-session decreases than SCFE mice ($g=2.67$, $p<0.0001$; $g=1.76$, $p<0.0001$, respectively). g = a standardized mean difference, computed as the FE1–FE3 contrast divided by the model residual SD. S1 mice used for this experiment, conventions as in Fig. 1. Supplement to **Fig. 2**.

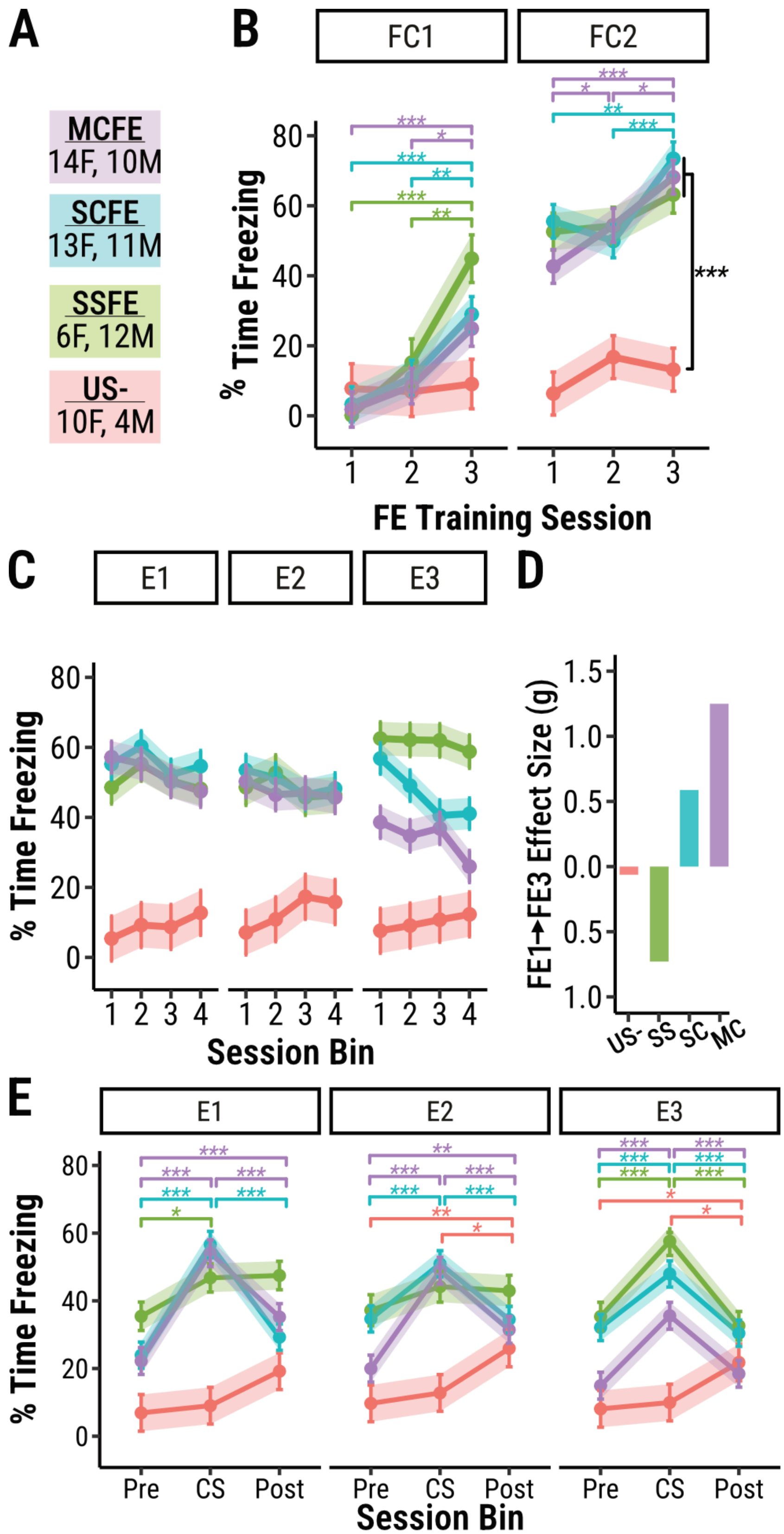
A
B
C
D
E
MCFE
14F, 10M
SCFE
13F, 11M
SSFE
6F, 12M
US-
10F, 4M
FC1
FC2
% Time Freezing
FE Training Session
E1
E2
E3
Session Bin
FE1→FE3 Effect Size (g)
US-
SS
SC
MC
Pre
CS
Post

Supplemental Figure 5. FC and FE behavior for Fos-mapping experiments.
(A) Sample sizes. **(B)** Cued fear conditioning (FC). All mice undergoing conditioning (MCFE, SCFE, SSFE) exhibited increased freezing across the CS–US presentations on FC1 and FC2. On FC2, the mice undergoing conditioning were freezing significantly more than the US- group (all $p<0.0001$). **(C)** Within-session extinction. **(D)** Standardized effect size (g) for the FE1→FE3 reduction in freezing. As previously reported, MCFE produced a larger across-session reduction in freezing ($g=1.25$, $p<0.0001$) than SCFE ($g=0.59$, $p=0.000155$). SSFE exhibited an increase in freezing from FE1→FE3, as mice in this condition did not receive CS presentations on FE1 ($g=-0.73$, $p<0.0001$). Finally, as expected, the unconditioned group did not show a difference in freezing from FE1 to FE3 ($g=-0.06$, $p=0.74$). **(E)** Comparison of Pre-, CS-, and Post-period freezing in the SSFE, SCFE, and MCFE groups. On FE1 and FE2, SSFE mice were exposed to the extinction context without CS presentations; freezing was therefore quantified during time periods matched to the designated CS epochs. With the exception of the Pre-to-CS transition on FE1 ($p<0.05$), freezing in SSFE mice did not differ across the Pre-, matched CS-, and Post-periods. In contrast, SCFE and MCFE mice, which received extinction training on these days, exhibited significantly greater freezing during the CS period relative to the Pre- and Post-periods. Critically, on FE3, the only session in which SSFE mice received CS presentations, SSFE mice exhibited clear CS-elicited freezing. TRAP2 x Ai14 mice used for this experiment, conventions as in Fig. 1. Supplement to **Fig. 3**.

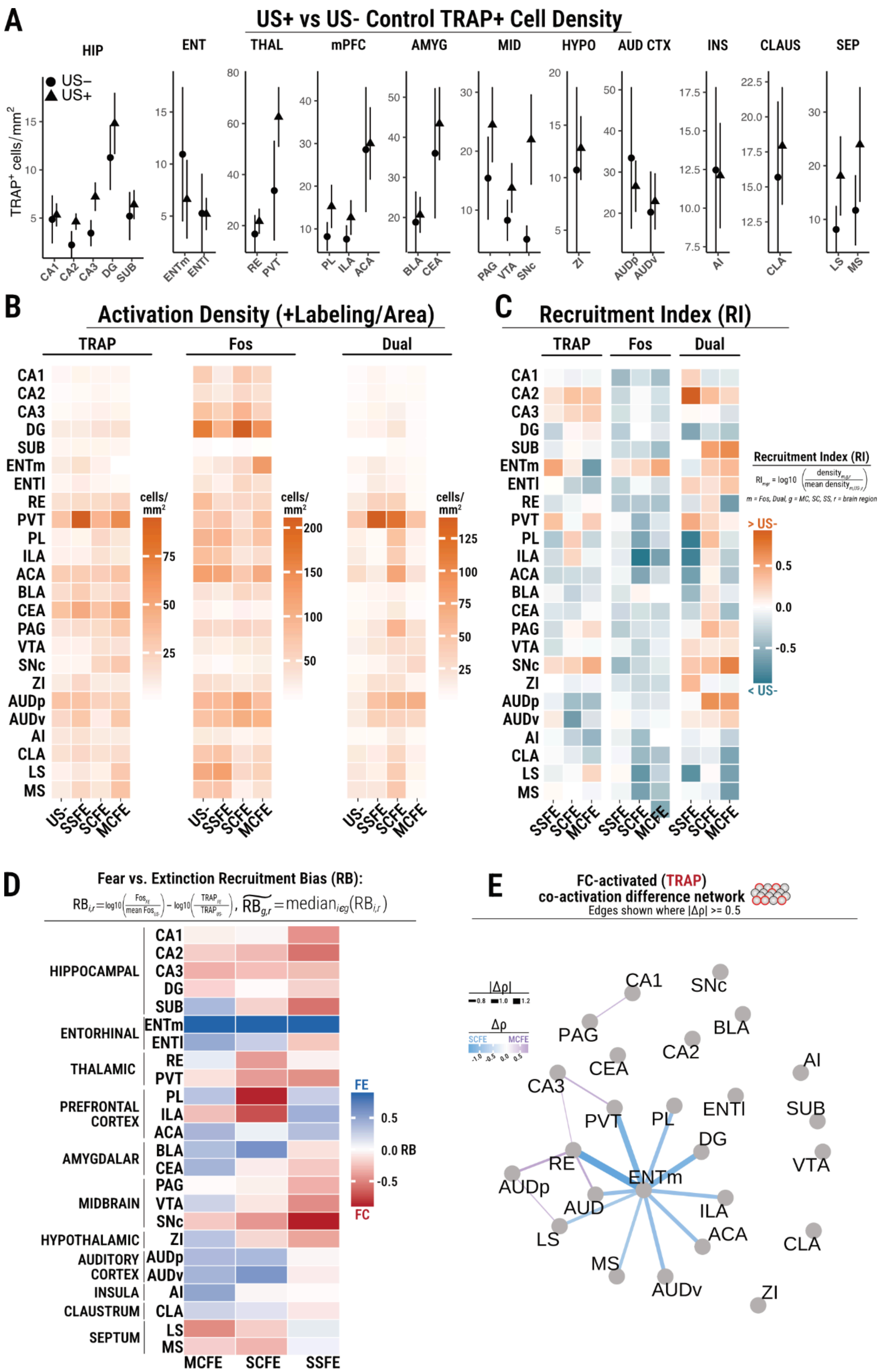
A
US+ vs US- Control TRAP+ Cell Density
HIP
ENT
THAL
mPFC
AMYG
MID
HYPO
AUD CTX
INS
CLAUS
SEP
US–
US+
TRAP+ cells/mm2
CA1
CA2
CA3
DG
SUB
ENTm
ENTl
RE
PVT
PL
ILA
ACA
BLA
CEA
PAG
VTA
SNc
ZI
AUDp
AUDv
AI
CLA
LS
MS
B
Activation Density (+Labeling/Area)
TRAP
Fos
Dual
cells/mm2
US-
SSFE
SCFE
MCFE
C
Recruitment Index (RI)
Recruitment Index (RI)
m = Fos, Dual, g = MC, SC, SS, r = brain region
> US-
< US-
D
Fear vs. Extinction Recruitment Bias (RB):
HIPPOCAMPAL
ENTORHINAL
THALAMIC
PREFRONTAL CORTEX
AMYGDALAR
MIDBRAIN
HYPOTHALAMIC
AUDITORY CORTEX
INSULA
CLAUSTRUM
SEPTUM
FE
FC
0.0 RB
MCFE
SCFE
SSFE
E
FC-activated (TRAP)
co-activation difference network
Edges shown where |Δρ| >= 0.5
|Δρ|
Δρ
SCFE
MCFE
AUD

Supplemental Figure 6. No differences in the magnitude of regional activation density. **(A)** Neuronal FC-labeling density (+TRAP cells/mm²) in mice exposed to the context and tones without footshock (US-, n=8) and mice fear conditioned with three CS–US pairings per day for two days (US+, n=25). TRAP labeling was induced on the second day of FC. Mice subsequently assigned to SSFE, SCFE, and MCFE were pooled for this analysis. US+ mice exhibited higher mean +TRAP density than US− mice in 20 of 24 regions; however, no regional comparisons survived FDR correction. This broad but statistically weak effect suggests that regional TRAP+ cell-density may not selectively capture shock-specific recruitment, but instead reflects multiple aspects of the conditioning experience, including context, sensory exposure, as well as handling and injection procedures shared by US- and US+ groups. **(B)** Activation density (+cell labeling/mm^2) by region, group, and type of labeling. No statistically significant group differences in activation density were observed in any region. For each animal and each brain region, area-weighted ("pooled") cell density was computed by summing cell counts across all sampled sections and dividing by the total annotated area. Briefly, within each animal and region, counts were pooled across sections (e.g. ∑Num-TRAP) and divided by the summed area (∑Area-mm²) to yield a single density value (cells mm^{-2}). These pooled densities provided one metric per animal per region, which were subsequently compared across experimental groups. Regional differences between groups were evaluated using Welch's t-tests, and effect sizes were quantified using Cohen's d. To account for multiple regional comparisons, false discovery rate (FDR; Benjamini-Hochberg) corrected p-values were also calculated. **(C)** To evaluate fear learning-related changes in activation, we also computed density per marker, group, and brain region normalized to the corresponding background US- control density and expressed as a log10 fold change (Recruitment Index). **(D)** To assess the sensitivity of the mean Recruitment Bias (RB) results shown in Fig 3D, we repeated the descriptive analysis using median RB. The median-based pattern was qualitatively similar to the mean-based analysis, including greater FE-related recruitment bias in MCFE. **(E)** Regional coactivation difference graph for FC (TRAP+) derived from inter-regional Spearman correlations (ρ) comparing FE groups ($\Delta\rho=\rho MCFE-\rho SCFE$, thresholded by $|\Delta\rho|>0.5$ for visualization. MCFE-SCFE differences in interregional Spearman correlations (Δρ) were less pronounced for TRAP+ labeling than Fos+ and Dual+ labeling shown in Fig 3E-F.

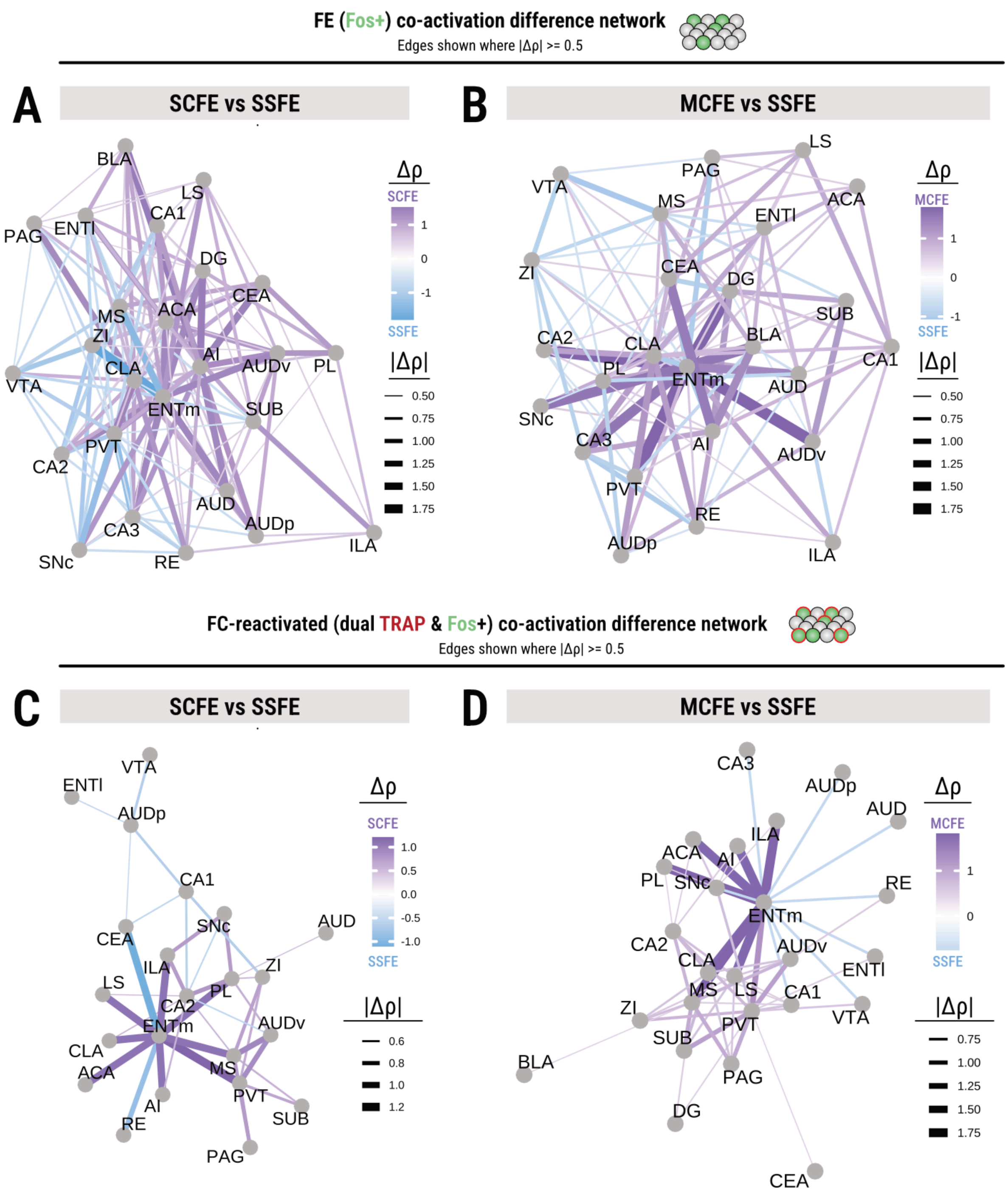

FE (Fos+) co-activation difference network
Edges shown where |Δρ| >= 0.5
A
SCFE vs SSFE
B
MCFE vs SSFE
Δρ
SCFE
SSFE
MCFE
|Δρ|
0.50
0.75
1.00
1.25
1.50
1.75
FC-reactivated (dual TRAP & Fos+) co-activation difference network
Edges shown where |Δρ| >= 0.5
C
SCFE vs SSFE
D
MCFE vs SSFE

Supplemental Figure 7. Co-activation difference networks for SCFE/MCFE and SSFE. FE-related inter-regional Spearman correlations (ρ) comparing SSFE to SCFE (**A**) and MCFE (**B**). The ENTm emerged as a key node for multiple-session extinction (SCFE degree=20, $\Sigma\Delta\rho$=7.20, MCFE degree=21, $\Sigma\Delta\rho$=13.60) and overall limited SSFE-biased coactivation (4/10 vs SCFE; 3/10 vs MCFE, see Supplementary Table 2). Similarly, when comparing FC-reactivated labeling between SSFE and SCFE (**C**) and MCFE (**D**), the ENTm emerged as a key hub (SCFE degree=10, $\Sigma\Delta\rho$=6.95, MCFE degree=17, $\Sigma\Delta\rho$=7.13) and multiple session extinction dominated coactivation bias (2/10 SSFE vs SCFE; 0/10 SSFE vs MCFE). Supplement to **Fig. 3**.

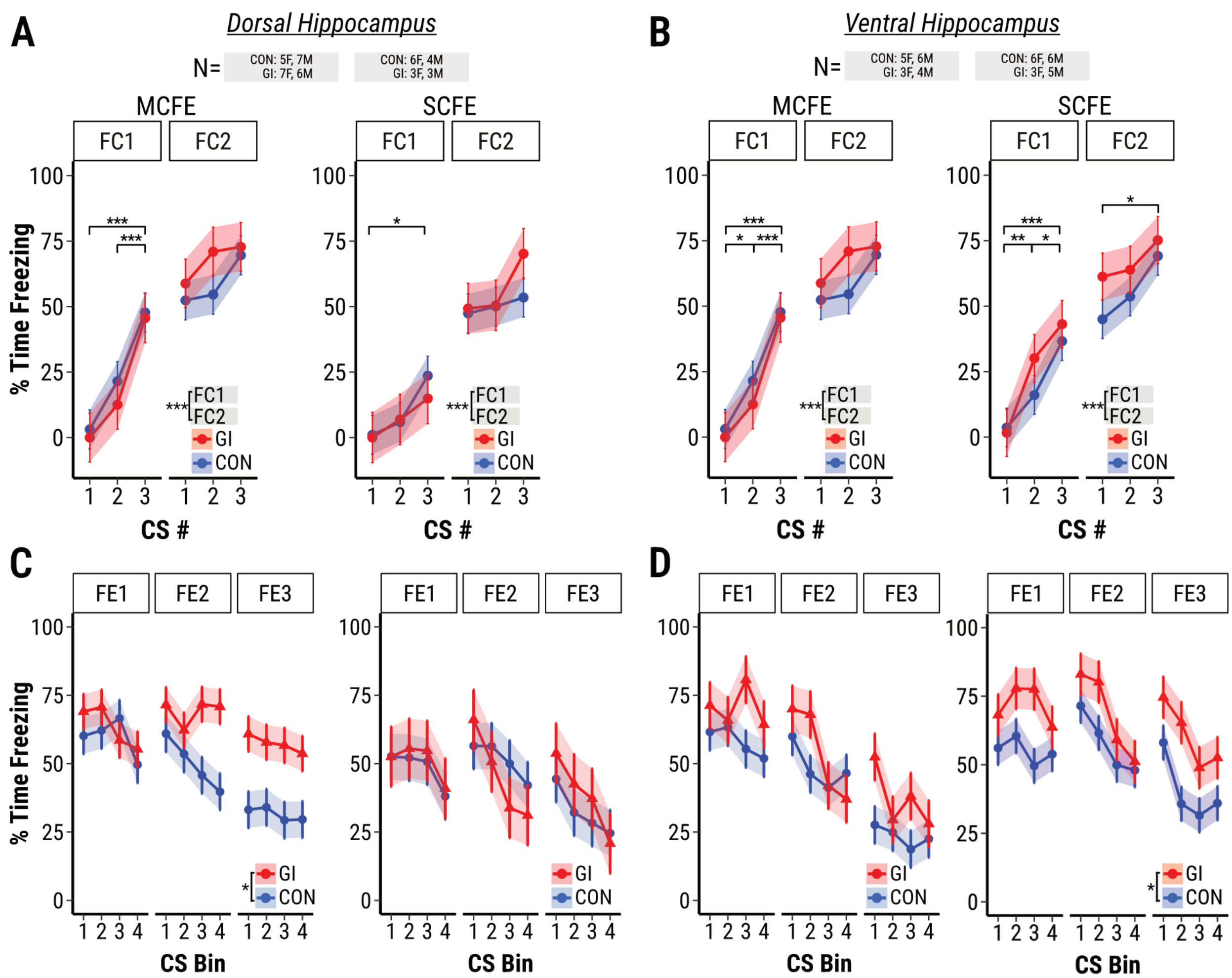

Supplemental Figure 8. FC and FE behavior during chemogenetic experiments.
(A-B) Cued fear conditioning (FC) for mice with dHP AAV injection **(A)** and vHP AAV injection **(B)**, stratified by MCFE (left) and SCFE (right). All mice increased freezing from FC1 to FC2 (all $p<0.0001$), regardless of viral targeting (dHP, vHP), condition (Gi, CON), and extinction group (SCFE, MCFE). **(C-D)** Within-session extinction for mice with dHP AAV injection **(C)** and vHP AAV injection **(D)**, stratified by MCFE (left) and SCFE (right). **(C)** For MCFE mice with dHP targeting (left), a main effect of bin was observed ($F[3,253]=3.6$, $p=0.0141$), consistent with B6 extinction behavior (**Fig. 1**). SCFE mice (right) also had a main effect of bin ($F[3,154]=13.98$, $p<0.0001$). **(D)** For mice with vHP targeting (left), a main effect of bin was observed for MCFE (left, $F[3,176]=8.55$, $p<0.0001$) and SCFE mice (right, $F[3,198]=17.59$, $p<0.0001$). B6 mice used for these experiments, conventions as in Fig. 1. Supplement to **Fig. 4**.

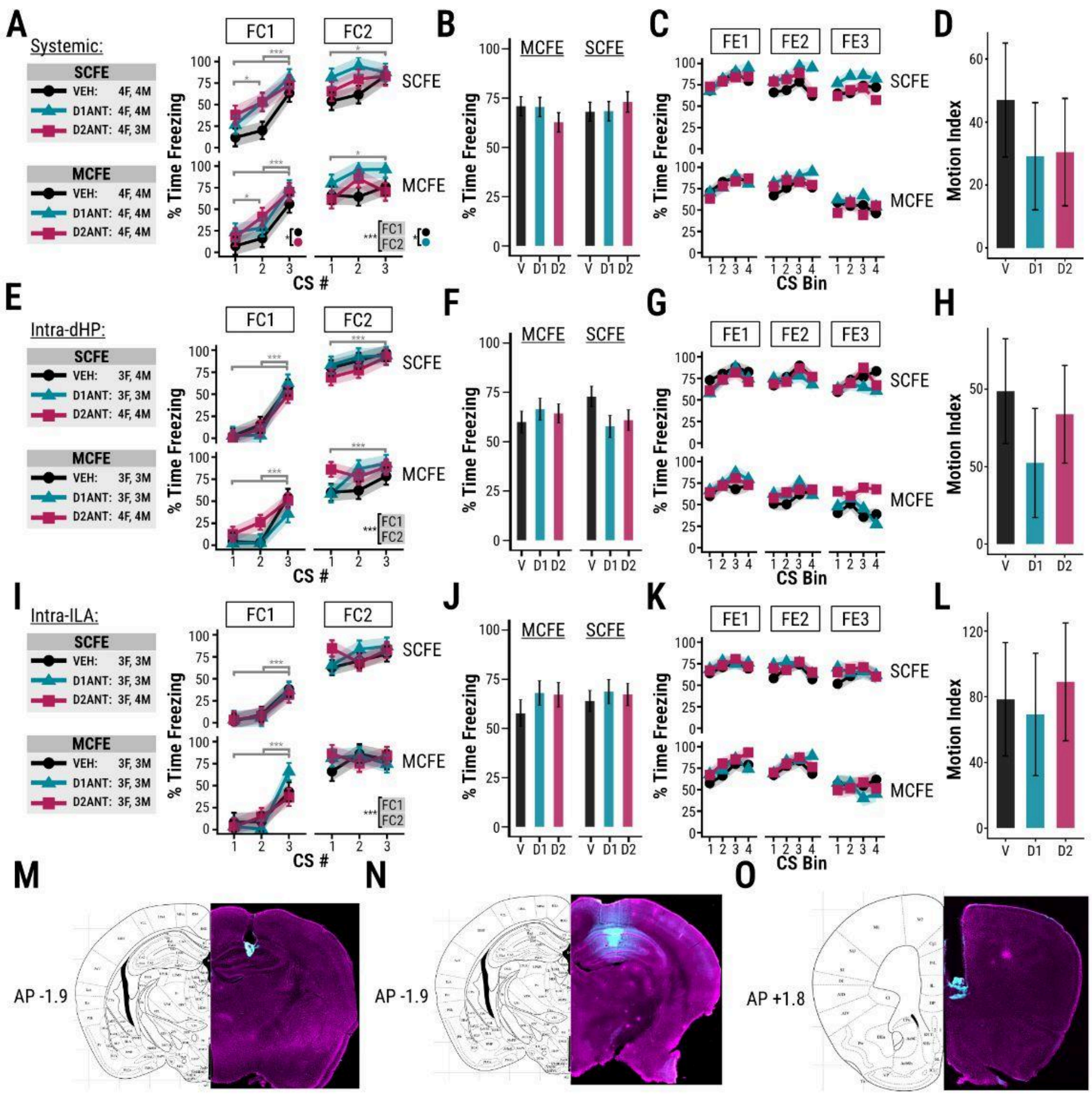

A
Systemic:
SCFE
VEH: 4F, 4M
D1ANT: 4F, 4M
D2ANT: 4F, 3M
MCFE
VEH: 4F, 4M
D1ANT: 4F, 4M
D2ANT: 4F, 4M
FC1
FC2
SCFE
MCFE
% Time Freezing
CS #
B
MCFE
SCFE
V D1 D2
C
FE1
FE2
FE3
CS Bin
D
Motion Index
V D1 D2
E
Intra-dHP:
SCFE
VEH: 3F, 4M
D1ANT: 3F, 3M
D2ANT: 4F, 4M
MCFE
VEH: 3F, 3M
D1ANT: 3F, 3M
D2ANT: 4F, 4M
F
G
H
I
Intra-ILA:
SCFE
VEH: 3F, 3M
D1ANT: 3F, 3M
D2ANT: 3F, 4M
MCFE
VEH: 3F, 3M
D1ANT: 3F, 3M
D2ANT: 3F, 3M
J
K
L
M
AP -1.9
N
AP -1.9
O
AP +1.8

Supplemental Figure 9. FC and FE behavior and infusion targeting for dopamine antagonism experiments.

(A) Cued fear extinction (FC) for systemic administration experiment. All mice increased freezing from FC1 to FC2, regardless of experimental group ($F[1,451]=104.35$, $p<0.0001$). Mice in the vehicle groups froze less than the D1Ant group ($t[40]=-2.78$, $p=0.025$). **(B)** Fear recall, systemic. No differences in freezing to the first three CS on FE1 observed between any groups (Context: $F[1,40]=0.2$, $p=0.653$; Condition: $F[2,40]=0.07$, $p=0.937$), indicating comparable conditioned fear at the start of extinction training. **(C)** Within-session fear extinction (FE), systemic. A main effect of bin was also observed ($F[3,451]=15.89$, $p<0.0001$). **(D)** Mean motion index during FE2 pre-tone period, systemic. No differences observed between drug conditions ($F[2,22]=1.36$, $p=0.278$). **(E)** Cued FC, intra-dHP. All mice increased freezing from FC1 to FC2, regardless of experimental group ($F[1,368]=501.81$, $p<0.0001$). **(F)** Fear recall, intra-dHP. No differences in freezing observed between any groups (Context: $F[1,33]=0$, $p=0.949$; Condition: $F[2,33]=0.38$, $p=0.684$). **(G)** Within-session FE, intra-dHP. A main effect of bin was observed ($F[3,374]=13.93$, $p<0.0001$). **(H)** Mean motion index, intra-dHP. No differences observed between drug conditions ($F[2,37]=1.91$, $p=0.163$). **(I)** Cued FC, intra-mPFC. All mice increased freezing from FC1 to FC2, regardless of experimental group ($F[1,352]=488.28$, $p<0.0001$). **(J)** Fear recall, intra-mPFC. No differences in freezing observed between any groups (Context: $F[1,31]=0.22$, $p=0.641$; Condition: $F[2,31]=0.86$, $p=0.432$). **(K)** Within-session FE, intra-mPFC. A main effect of bin was observed ($F[3,287]=10.17$, $p<0.0001$). **(L)** Mean motion index, intra-mPFC. No differences observed between drug conditions ($F[2,36]=0.3$, $p=0.739$). **(M)** Example dHP infusion targeting, visualized via fluorescently-labeled CTB marker. **(N)** Example dHP infusate spread estimate, visualized via sodium fluorescein infusion. **(O)** Example mPFC infusion targeting, visualized via fluorescently-labeled CTB marker. Mean motion index was calculated by *VideoFreeze* as the average frame-to-frame pixel change across the recording session [1]. S1/B6 hybrids used for these experiments, conventions as in Fig. 1. Supplement to **Fig. 5**.

Supplementary Table 1. Contextual features of behavioral chambers

Context	Floor	Walls	Scent	Lighting
A	Thin stainless steel rods	Aluminum	30% distilled white vinegar in water	No overhead white light
B	Smooth white acrylic	Curved white acrylic	75% ethanol	Overhead white light
C	Smooth black acrylic	Triangular black acrylic	75% ethanol + vanilla essential oil	Overhead white light
D	Lightly textured blue foam surface	Curved green/white striped plastic	75% ethanol + lavender essential oil	Overhead white light
E	Black/white polka dot textured plastic	Curved black/white polka dot textured plastic	75% ethanol + orange essential oil	Overhead white light
F	Blue rubber mat with circular pads	Curved orange plastic	75% ethanol + almond essential oil	Overhead white light

Supplementary Table 2. Top Differential Nodes for FE-activation and FC-reactivation

Top differential coactivation nodes<br>MCFE–SCFE networks; edges shown where |Δρ| ≥ 0.5						
**Region**	**Degree**	**Σ|Δρ|**	**ΣΔρ**	**MCFE-biased**	**SCFE-biased**	**Net bias**
**FE Activation (Fos+)**						
ENTm	10	7.20	2.40	7	3	MCFE
PL	9	7.78	-6.58	1	8	SCFE
ACA	7	5.90	-5.90	0	7	SCFE
AUD	7	5.61	-4.56	1	6	SCFE
ZI	6	5.08	-0.45	3	3	SCFE
PVT	6	4.25	1.77	4	2	MCFE
ILA	6	3.87	-2.67	1	5	SCFE
AUDv	5	4.22	-4.22	0	5	SCFE
MS	5	3.30	-1.06	2	3	SCFE
SUB	5	3.10	-0.70	2	3	SCFE
**FC Reactivation (Dual TRAP & Fos+)**						
CA1	11	6.98	6.98	11	0	MCFE
ENTm	9	6.38	5.00	8	1	MCFE
PVT	4	2.65	2.65	4	0	MCFE
CEA	4	2.52	2.52	4	0	MCFE
AUD	4	2.30	0.91	3	1	MCFE
CA2	4	2.29	2.29	4	0	MCFE
AUDp	3	2.03	2.03	3	0	MCFE
LS	3	1.92	1.92	3	0	MCFE
VTA	3	1.91	1.91	3	0	MCFE
RE	3	1.77	1.77	3	0	MCFE
**Degree** = Number of suprathreshold (Δρ = 0.5) differential edges connected to that region						
**Σ|Δρ|** = Unsigned total differential edge strength (ρMCFE–ρSCFE)						
**ΣΔρ** = Signed strength (ρMCFE–ρSCFE)						
**MCFE-biased / SCFE-biased** = Number of connected edges where Δρ is positive or negative						

Supplementary Table 3. Differential co-activation between SCFE and SSFE

Top differential coactivation nodes<br>SCFE–SSFE networks; edges shown where |Δρ| ≥ 0.5						
**Region**	**Degree**	**Σ|Δρ|**	**ΣΔρ**	**SCFE-biased**	**SSFE-biased**	**Net bias**
**FE Activation (Fos+)**						
ENTm	20	22.40	7.20	12	8	SCFE
CLA	18	17.05	15.85	17	1	SCFE
PVT	17	14.74	-5.09	5	12	SSFE
ACA	17	14.60	14.60	17	0	SCFE
MS	15	12.07	-3.63	6	9	SSFE
ZI	14	12.61	-4.84	5	9	SSFE
AI	13	15.37	13.37	12	1	SCFE
CA3	13	10.68	-1.32	5	8	SSFE
DG	12	9.53	8.52	11	1	SCFE
CA1	12	8.98	1.43	7	5	SCFE
**FC Reactivation (Dual TRAP & Fos+)**						
ENTm	10	10.95	6.95	8	2	SCFE
CA2	9	5.46	2.04	6	3	SCFE
MS	6	4.69	4.69	6	0	SCFE
PL	6	4.32	4.32	6	0	SCFE
PVT	5	4.13	4.13	5	0	SCFE
AUDv	4	2.98	1.89	3	1	SCFE
ZI	4	2.55	1.25	3	1	SCFE
CA1	4	2.46	-2.46	0	4	SSFE
AUDp	4	2.33	-2.33	0	4	SSFE
ILA	3	2.64	2.64	3	0	SCFE
**Degree** = Number of suprathreshold (Δρ = 0.5) differential edges connected to that region						
**Σ|Δρ|** = Unsigned total differential edge strength (ρSCFE–ρSSFE)						
**ΣΔρ** = Signed strength (ρSCFE–ρSSFE)						
**SCFE-biased / SSFE-biased** = Number of connected edges where Δρ is positive or negative						

Supplementary Table 4. Differential co-activation between MCFE and SSFE

Top differential coactivation nodes<br>MCFE–SSFE networks; edges shown where $|\Delta\rho| \geq 0.5$						
**Region**	**Degree**	**$\Sigma|\Delta\rho|$**	**$\Sigma\Delta\rho$**	**MCFE-biased**	**SSFE-biased**	**Net bias**
**FE Activation (Fos+)**						
ENTm	21	24.00	13.60	15	6	MCFE
CLA	19	16.55	14.55	18	1	MCFE
DG	14	10.77	7.83	12	2	MCFE
MS	13	9.66	-0.88	6	7	SSFE
AI	11	10.07	8.07	10	1	MCFE
CEA	11	9.21	4.59	8	3	MCFE
AUD	11	8.25	4.14	8	3	MCFE
BLA	10	8.72	4.82	7	3	MCFE
RE	10	7.04	-0.54	4	6	SSFE
PL	10	6.74	-0.27	5	5	SSFE
**FC Reactivation (Dual TRAP & Fos+)**						
ENTm	17	20.48	7.13	8	9	MCFE
PVT	12	9.66	9.66	12	0	MCFE
MS	9	7.79	7.79	9	0	MCFE
CLA	7	5.92	5.92	7	0	MCFE
LS	7	5.59	5.59	7	0	MCFE
CA2	7	4.73	4.73	7	0	MCFE
AUDv	6	4.55	3.07	5	1	MCFE
SNc	6	3.68	2.19	5	1	MCFE
CA1	5	3.13	1.65	4	1	MCFE
SUB	4	3.51	3.51	4	0	MCFE
**Degree** = Number of suprathreshold ($\Delta\rho$ = 0.5) differential edges connected to that region						
**$\Sigma|\Delta\rho|$** = Unsigned total differential edge strength (ρMCFE–ρSSFE)						
**$\Sigma\Delta\rho$** = Signed strength (ρMCFE–ρSSFE)						
**MCFE-biased / SSFE-biased** = Number of connected edges where $\Delta\rho$ is positive or negative						

Supplemental Information

Methods and Materials

Mice

Founder mice were obtained from Jackson Laboratories and bred in our colony. All mice were housed by sex in cages of 2-5 subjects with 24/7 access to food and water *ad libitum*. Mice were on a 12-hr on/off light cycle and all experiments were conducted during the light-on phase. Prior to fear conditioning, mice were randomly assigned to SCFE (single context fear extinction) or MCFE (multiple context fear extinction) groups of about the same size with roughly equal numbers of males and females in each group. Exact numbers of mice are provided in figure captions. All procedures performed using mice were approved by and carried out in accordance with the Williams College Institutional Care and Use Committee (IACUC).

Experiment 1 (Fig 1A-F, S1D-G): Male and female 129S1/SvImJ (Stock #002448), C57BL/6J (Stock #000664) mice were used to determine whether the effect of multiple-context extinction learning generalized across strains that differ in stress susceptibility and extinction learning [2,3].

Experiment 2-3 (Fig 1G-I, S2A-C; Fig 2A-E, S3A-F, S4A-G): To assess boundary conditions of MCFE, these studies focused on the 129S1/SvImJ (Stock #002448), which exhibit impaired extinction learning and greater susceptibility to fear relapse. This provides a stringent background in which to test whether contextual variation improves extinction and reduces context-dependent relapse.

Experiment 4 (Fig 3A-H; S5A-E, S6A-E, S7A-D): Learning-related neural recruitment was assessed using TRAP2 x Ai14 mice generated by crossing Fos-iCre-ER mice (Stock #030323) with Cre-dependent tdTomato reporter Ai14 (Stock #007914), as previously described [4]. Both transgenic strains are maintained in a C56BL/6J background. The genetic background of this experiment, therefore, reflected the requirements of the activity tagging approach.

Experiment 5 (Fig 4A-F; S8A-D): The functional role of the hippocampus was tested in the C57BL/6J strain because these experiments were motivated by the Fos-mapping results from Experiment 4 and was designed to test causal significance of the brain region identified in that experiment.

Experiment 6 (Fig 5A-I; S9A-O): To test dopamine receptor involvement in the dorsal hippocampus and mPFC, we used F1 hybrid 129S1/SvImJ (Stock #002448) X C57BL/6J (Stock #000664) mice. We selected this cross because the parental strains showed markedly different renewal phenotypes in our preceding experiments. Our objective was to use the F1 cross to test the dopamine-dependent mechanism in a genetically distinct population spanning these parental backgrounds, ultimately enabling us to compare animals with varying levels of renewal from the same genetic background. The F1 cross nevertheless uniformly exhibited little renewal, more closely resembling the C57BL/6J phenotype. Accordingly, all animals were combined for analyses.

Fear conditioning apparatus
Fear conditioning was conducted in 9.5 x 12 x 8.25” chambers (#VFC2-USB-M, Med-Associates, Fairfax, Vermont) housed in 24.25” x 22” x 28.75” sound attenuated boxes. Each chamber had clear acrylic backs and doors, aluminum sides, stainless steel rod floors (spaced 0.31” apart), stainless steel drop pans, and was illuminated by overhead near infrared (940 nm) light. Foot shocks were administered through the rods via solid state shock scramblers. Behavior was recorded at a frame rate of 30 Hz using a monochrome front-facing camera equipped with a near-infrared pass filter. *VideoFreeze* (Med-Associates) running on a desktop PC was used to acquire images and to program delivery of all stimuli. Freezing behavior was operationalized as complete immobility except for movement required for breathing. It was quantified using automated scoring via *VideoFreeze*, with a motion threshold of 18 and a minimum bout duration of 1 s. To ensure that the automated scoring accurately captured differences in freezing, using stratified random sampling, we selected 40 videos (20 from FC2, 20 from FE3 sessions, evenly distributed across experiments and across SCFE/MCFE and female/male mice) and manually scored freezing behavior throughout the entire session using BORIS (Behavioral Observation Research Interactive Software) [5]. We then compared manually scored time freezing during CS delivery and pre- and post-tone periods with *VideoFreeze*-derived freezing scores for these same time periods. *VideoFreeze* scores were strongly correlated with manual freezing scores (overall r=0.88), validating automated freezing quantification, although *VideoFreeze* showed a modest tendency to estimate higher freezing scores as compared to manual scoring (**S1 A-C**).

Cued fear conditioning
Mice were fear-conditioned once per day over the course of two consecutive days. The protocol consisted of three tones (4 kHz), twenty-five seconds in length used as conditioned stimuli (CS), which were immediately followed by a two second 0.7-0.75 mA footshock (unconditioned stimulus, US). The protocol length totaled 9 minutes and 14 seconds. Both days of conditioning occurred in context A. Once the protocol terminated, the mice were removed from the chambers and placed into their home cages. In between cohorts, the chambers were wiped down with the same solutions used to produce the ambient scent.

Fear extinction, recall tests, and fear reinstatement
After fear conditioning, mice underwent up to 3 consecutive days of fear extinction consisting of 12 unreinforced CS-tones with inter-stimulus intervals ranging from 5 to 22s. For mice undergoing single context fear extinction (SCFE), each training session over the 3 days was conducted in context B, while for mice undergoing multiple context fear extinction (MCFE), each training session was conducted in a unique context (B-D). Recall tests were carried out 2 and 30 days following fear extinction and consisted of the presentation of 4 unreinforced CS-tones. For fear reinstatement experiments, mice were re-exposed to three US-footshocks in Context A following fear extinction training.

Dual labeling: Fos neuronal tagging and immunofluorescence

Fear conditioning and fear extinction were carried out as previously described with several modifications: First, to permit robust tdTomato expression, a 7-d interval was incorporated between FC2, when TRAP-mediated Cre expression was induced, and the start of fear extinction training. Second, two additional control groups were included: Single session FE (SSFE) to index neuronal recruitment during early FE acquisition, and a no-US control (US-), to index background Fos labeling. SSFE mice underwent the same fear conditioning as SCFE and MCFE, but received context exposure alone during FE days 1-2 and were exposed to the CS on FE3, corresponding to the third extinction session for SCFE and MCFE. US- mice underwent the same context and tone exposures as the other groups, but never received a footshock US. For both SSFE and US- groups context exposure was counterbalanced such that half of the mice received the same context across sessions (B, B, B), whereas the remaining mice experienced distinct contexts (B, C, D).

Dual fos labeling of active cell populations was achieved by combining the TRAP2 system and Fos immunolabeling [4]. Recombination was induced via injection with tamoxifen (B5965-5000, APExBIO). TAM (30 mg/ml) was dissolved directly in corn oil at 50 °C and filtered through a sterile syringe filter (0.2 μm PES, Millipore Sigma) [6]. To minimize non-specific IEG activity, mice were maintained in a holding room adjacent to the animal testing room and were otherwise left undisturbed for a full 24 hour period surrounding the TAM injection. Fear learning-related activity-dependent neuronal tagging was induced by a single intraperitoneal injection of TAM (50 mg/kg) administered immediately after the cued fear conditioning session on day 2 in TRAP2 x Ai14 mice. A 7-d interval preceded the start of extinction training to permit tdTomato reporter expression. Tissue was collected 10-d after TRAP induction, 90 minutes after the last extinction session.

To label active cell populations related to extinction learning, mice were deeply anesthetized with avertin (500 mg/kg) and transcardially perfused with 1× PBS, followed by 4% paraformaldehyde in 1× PBS ~90 m after the last extinction session. Extracted brains were post-fixed overnight at 4 °C in 4% paraformaldehyde. For immunohistochemistry, 100-μm coronal sections were collected on a vibratome, washed in 1× PBS and blocked at room temperature for 1hr in 10% normal goat serum (NDS) in 1× PBS with 0.3% Triton X-100 (PBST). Sections were incubated with primary antibodies—1:1,000 rabbit anti-Fos (Cell Signaling #2250), for 48hr at 4 °C. Sections were rinsed in 1× PBST and incubated in secondary antibodies (1:1000 goat anti-rabbit Alexa 647) in 1× PBST for 1hr at room temperature. Sections were then rinsed in 1× PBST and counterstained with DAPI (3 uM, Invitrogen, #D1306), mounted onto slides, and coverslipped with antifade reagent in glycerol (90% Glycerol, 0.5% N-propyl gallate, 20 mM Tris, pH 8.0)

Imaging and quantification

Fluorescence images were acquired using an Olympus VS200 slide-scanning epifluorescence microscope equipped with a 10x objective (UPLXAPO, NA 0.40), an EM-CCD camera (Orca Fusion), LED illumination (X-Cite Xylis) and optical filters. Optically stitched images of brain sections were produced for TRAP (tdTomato; filters, Ex 605/70; BS 565; Em 545/25) and Fos labeling (647 immunofluorescence; filters:Ex 635/18, BS652, Em 680/42). To align imaged

brains to the Allen Mouse Brain Common Coordinate Framework, sections were adjusted for anterior-posterior position, angle, rotation, and scale using a deep neural network (DeepSlice [7,8]).

Cell Classification
Fluorescence images containing Fos immunolabeling (Cy5) and TRAP-derived tdTomato fluorescence (Cy3) were exported as composite two-channel TIFF images and analyzed in QuPath (v0.5.5 [9]). Cellular objects were detected using the StarDist extension with the default fluorescence model [10]. A permissive detection threshold was used during StarDist segmentation to maximize sensitivity and ensure that potentially labeled cells were retained for subsequent classification. Following object detection, fluorescence-positive cells were identified using a local region-of-interest (ROI)-based thresholding procedure. Representative ROIs were sampled from each image, and thresholded using 95th percentile pixel intensity for the ROI and a fixed offset of (+1600 globally, +1400 for hippocampal regions). Fluorescence threshold was calculated from the local fluorescence intensity distribution of a composite tdTomato (TRAP) and Fos image. The resulting threshold was then used to filter StarDist detections, retaining only objects with fluorescence intensity exceeding the automatically determined threshold.

To distinguish true Fos-positive and tdTomato-positive cells from autofluorescence, tissue artifacts, and segmentation errors, machine-learning classifiers were trained separately for each fluorophore using QuPath's object classification framework. Training annotations were generated from representative sections spanning the range of staining intensities observed across the dataset and were iteratively refined until classifier performance reliably distinguished labeled Fos- and/or tdTomato-positive cells from non-specific detections. The classifiers incorporated object-level intensity, morphological, and texture features derived from the segmented StarDist objects. Objects classified as Fos- and/or tdTomato-positive were exported for quantitative analysis. Counts of Fos-positive, tdTomato-positive, and dual-labeled cells were subsequently quantified within atlas-defined brain regions and used for all downstream analyses.

Positive cell detection density: For each animal and each brain region, we computed an area-weighted ("pooled") cell density by summing cell counts across all sampled sections and dividing by the total annotated area. Briefly, for each ROI the number of $TRAP^+$, Fos^+, and dual-labeled cells was exported from QuPath along with its area in μm^2. Within each animal and region, counts were then pooled across sections (e.g. ∑Num-TRAP) and divided by the summed area (∑Area-mm^2) to yield a single density value (cells mm^{-2}). These pooled densities and overlap fractions provided one metric per animal per region, which were subsequently compared across experimental groups (**S6A**).

Recruitment Score: To account for non-learning related neuronal tagging, one group of mice (US-) underwent the same behavioral procedures as the experimental groups (ie tone and context exposure) but did not receive footshock pairings. For each brain region, marker-specific recruitment was calculated as the $\log_{10}$ fold-change in density relative to the mean density of the background US- control group. Recruitment indices were computed separately for TRAP, Fos

and dual-labeled density at the animal level, then averaged within each group and region for visualization. Positive values indicate greater recruitment than US- controls, whereas negative values indicate lower recruitment than US- controls (**S6C**). Recruitment Index values were expressed as log10 fold-changes relative to the corresponding US- mean density in order to place changes (increases or decreases) on a symmetric scale centered at zero and to facilitate comparison across labels.

Fos recruitment bias: The recruitment score for Fos-labeling was used as an index of extinction-session recruitment, whereas the TRAP recruitment score was used as an index of fear-conditioning recruitment. An Extinction-Fear Recruitment Bias was then calculated by subtracting the TRAP recruitment score from the Fos recruitment score. Positive values indicate greater extinction-associated recruitment relative to fear-associated recruitment, whereas negative values indicate greater fear-associated recruitment relative to extinction-associated recruitment. Scores were then averaged across animals within each experimental group and brain region for visualization as a heatmap.

Reactivated fear and extinction-related co-variation: Inter-regional co-activation was assayed by computing pairwise Spearman correlations (ρ) of regional Fos+ and dual-labeled densities across animals, and MCFE–SCFE difference networks were visualized as $\Delta\rho=\rho MCFE-\rho SCFE$. From these, we quantified several parameters such as the number of suprathreshold edges per node ($|\Delta\rho|\geq0.50$), signed as well as total unsigned differential edge strength ($\Sigma\Delta\rho$, $\Sigma|\Delta\rho|$).

To assess condition-specific organization of reactivated-cell density across neuroanatomical systems, we performed a parent system-level correlation analysis. Regions were assigned to *a priori* neuroanatomical systems, including hippocampus (CA1, CA2, CA3, DG, SUB), medial prefrontal cortex (PL, ILA, ACA), amygdala (BLA, CEA), thalamus (RE, PVT), entorhinal cortex (ENTl, ENTm) and midbrain (PAG, VTA, SNc). For each extinction condition, animal-by-region matrices were generated using Fos- and reactivated-cell density values. Spearman correlations were then computed across animals between all region pairs within each condition. For each anatomical systems pair, we summarized the network score as the mean Spearman correlation among all region pairs belonging to that anatomical systems pair. For within-anatomical systems scores, we averaged only the unique pairwise correlations among regions.

To estimate the stability of anatomical systems-level correlation scores, we used nonparametric bootstrapping at the animal level. Within each condition, animals were resampled with replacement, the animal-by-region matrix was reconstructed, and anatomical systems-level correlation scores were recomputed for each bootstrap iteration. This procedure was repeated 5,000 times per condition. Bootstrap means and 95% confidence intervals were computed from the resulting bootstrap distributions. Anatomical systems-level correlations were considered stable and positive when the 95% bootstrap confidence interval excluded zero in the positive direction.

To test whether stable positive anatomical systems-level correlations exceeded values expected under a null model in which regional Fos- and reactivated-cell densities were unrelated across animals, we performed within-group permutation testing. Within each condition, values were

independently shuffled across animals for each region, preserving the marginal distribution of each region while disrupting cross-region covariance structure. Anatomical systems-level correlation scores were recomputed after each shuffle, generating a null distribution for each group and anatomical systems pair. Empirical one-sided p-values were calculated as the proportion of permuted scores greater than or equal to the observed score, with a +1 correction applied to the numerator and denominator. Two-sided p-values were also computed using the absolute observed and permuted values. False discovery rate correction was applied across tested group × anatomical systems-pair combinations using the Benjamini–Hochberg procedure.

Chemogenetics

Mice underwent stereotaxic surgery to infuse purified adeno-associated virus (AAV) to transduce an inhibitory designer receptor exclusively activated by designer drug (DREADD, AAV5-CaMKII-hM4D(Gi)-IRES-mCitrine, “GI”), or a control fluorescence marker (AAV5-CaMKII-mCherry, “CON”). These procedures were performed 2-4 weeks prior to behavioral experimentation. In brief, mice were maintained under anesthesia with 1-3% isoflurane and secured in a stereotaxic frame, and craniotomies were performed to inject AAV into dorsal (AP -1.9; ML +/- 1.5; DV -1.8; 500 nl) or ventral (AP -3.16; ML +/-3.0; DV -3.85, -3.5, -3.25; 200 nl/site) hippocampus [11]. After the surgery, mice were injected subcutaneously with the analgesic carprofen (5 mg/kg) in sterile saline (0.9%). All mice, independent of AAV condition, were habituated to handling and scruffing for 7 days prior to the start of behavioral experimentation. On extinction session day 2, 30 minutes before the start of training, mice were injected with DREADD agonist Compound 21 (C21, 1 mg/kg), which does not undergo back-metabolism to clozapine [12].

Drug administration

Mice received either *intraperitoneal* (*i.p.*) or *intracerebral* (*i.c.*) administration of SCH23390, raclopride, or vehicle (saline) prior to the FE2 behavioral session. *i.p.* injections of SCH23390 (0.05 mg/kg) and vehicle were delivered 15 min before behavior, whereas raclopride (0.3 mg/kg) was given 30 min before behavior.

i.c. infusions were delivered through 26-ga bilateral cannulae (PlasticsOne) either implanted to target the ILA within the mPFC (AP +1.8; ML +/- 0.25; DV -2.4) or dHP (AP - 1.9; ML +/- 1.5; DV -1.3) [13–16]. At least one week of recovery following cannula implantation was allowed, during which mice were singly housed but received 30 min of daily glove handling, socialization with other animals, and enrichment with running wheels, tubes, and golf balls. On E2, dust caps and dummy cannulae were removed from the guide cannulae, and the infusion tubing was prepared by connecting two 10 μL syringes to 33-ga bilateral internal cannulae that extended 0.5 mm below the guide cannulae (PlasticsOne) with polyethylene tubing. The entire length of tubing was filled with the drug, and the internal cannulae were lowered into the guide cannulae. All infusions (SCH23390: 3 μg/side; raclopride: 2.5μg/side) were delivered at a rate of 0.2 μL/min, with a total volume of 0.5 μL/side delivered to dHP and 0.25 μL/side delivered to ILA. The internal cannulae remained in place for 5min following completion of the infusion to prevent

backflow. The mouse was then returned to its homecage for 15 (SCH23390) to 20 min (raclopride) before starting behavioral experimentation [10,11].

At the conclusion of the five-day experiment, fluorescently-labeled cholera toxin B (CTB-488) was infused intracerebrally according to the above procedure such that the cannulae termini could be visualized and aligned to the Paxinos and Franklin Mouse Brain Atlas post perfusions, sectioning, and imaging (**S9 M,O**) [17]. Because raclopride infusion in dHP was found to affect extinction learning, several dHP vehicle animals received infusions of sodium fluorescein in order to better estimate infusate spread, as fluorescein's smaller molecular size (376 Da) allows diffusion properties more comparable to those of raclopride (346 Da) than a larger tracer such as CTB (11.6 kDa, **S9N**).

Statistical analyses
All data and relevant code is available on figshare: https://doi.org/10.6084/m9.figshare.33000362. Additionally, complete statistical results are available in the **Supplementary Statistics File**. Behavioral outcomes were analyzed with linear mixed-effects models that included a random intercept for subject (Animal) and fixed factors matched to each design (e.g., group, CS, day, and their interactions) [18]. Pre- and post-CS periods were excluded. Categorical predictors were effects-coded (sum-to-zero) so that main effects represent deviations from the grand mean; ordered factors (e.g., CS position, day) were additionally examined for trend components (linear, quadratic) where appropriate. Omnibus (Type III) tests were used to evaluate main effects and interactions, with Satterthwaite-approximated degrees of freedom. When interactions were significant, we conducted simple-effects tests and reported Holm-adjusted pairwise contrasts; main-effect post hocs were only performed when no higher-order interaction involving that factor was significant. For visualization, we plotted estimated marginal means (EMMs) with standard errors (SE). Unless noted, tests were two-tailed with $\alpha = .05$. Where reported, the standardized effect size g denotes the specified model contrast divided by the model's residual standard deviation.

Supplemental References

1. Anagnostaras SG, Wood SC, Shuman T, Cai DJ, LeDuc AD, Zurn KR, et al. Automated Assessment of Pavlovian Conditioned Freezing and Shock Reactivity in Mice Using the Video Freeze System. Front Behav Neurosci. 2010;4:158.

2. Camp M, Macpherson K, Lederle L, Graybeal C, Gaburro S, Debrouse L, et al. Genetic strain differences in learned fear inhibition associated with variation in neuroendocrine, autonomic, and amygdala dendritic phenotypes. Neuropsychopharmacol Official Publ Am Coll Neuropsychopharmacol. 2012;37:1534–1547.

3. Singewald N, Holmes A. Rodent models of impaired fear extinction. Psychopharmacology. 2018;236:21–32.

4. DeNardo LA, Liu CD, Allen WE, Adams EL, Friedmann D, Fu L, et al. Temporal evolution of cortical ensembles promoting remote memory retrieval. Nat Neurosci. 2019;22:460–469.

5. Friard O, Gamba M. BORIS: a free, versatile open-source event-logging software for video/audio coding and live observations. Methods Ecol Evol. 2016;7:1325–1330.

6. Donocoff RS, Teteloshvili N, Chung H, Shoulson R, Creusot RJ. Optimization of tamoxifen-induced Cre activity and its effect on immune cell populations. Sci Rep. 2020;10:15244.

7. Carey H, Pegios M, Martin L, Saleeba C, Turner AJ, Everett NA, et al. DeepSlice: rapid fully automatic registration of mouse brain imaging to a volumetric atlas. Nat Commun. 2023;14:5884.

8. Cabrera S, Vachoud N, Maciel R, Breuilly M, Desmercieres S, Meyer-Dilhet G, et al. Establishment of an optimized and automated workflow for whole brain probing of neuronal activity. bioRxiv. 2025:2024.09.16.611953.

9. Bankhead P, Loughrey MB, Fernández JA, Dombrowski Y, McArt DG, Dunne PD, et al. QuPath: Open source software for digital pathology image analysis. Sci Rep. 2017;7:16878.

10. Schmidt U, Weigert M, Broaddus C, Myers G. Medical Image Computing and Computer Assisted Intervention – MICCAI 2018, 21st International Conference, Granada, Spain, September 16-20, 2018, Proceedings, Part II. Lect Notes Comput Sci. 2018:265–273.

11. Stein RE, Kaplan JS, Li J, Catterall WA. Hippocampal deletion of NaV1.1 channels in mice causes thermal seizures and cognitive deficit characteristic of Dravet Syndrome. Proceedings of the National Academy of Sciences of the United States of America. 2019;116:16571–16576.

12. Thompson KJ, Khajehali E, Bradley SJ, Navarrete JS, Huang XP, Slocum S, et al. DREADD

Agonist 21 Is an Effective Agonist for Muscarinic-Based DREADDs in Vitro and in Vivo. ACS Pharmacol Transl Sci. 2018;1:61–72.

13. Hikind N, Maroun M. Microinfusion of the D1 receptor antagonist, SCH23390 into the IL but not the BLA impairs consolidation of extinction of auditory fear conditioning. Neurobiol Learn Mem. 2008;90:217–222.

14. Mueller D, Bravo-Rivera C, Quirk GJ. Infralimbic D2 Receptors Are Necessary for Fear Extinction and Extinction-Related Tone Responses. Biol Psychiatry. 2010;68:1055–1060.

15. Corcoran KA, Maren S. Hippocampal Inactivation Disrupts Contextual Retrieval of Fear Memory after Extinction. J Neurosci. 2001;21:1720–1726.

16. Corcoran KA, Maren S. Factors regulating the effects of hippocampal inactivation on renewal of conditional fear after extinction. Learning & Memory. 2004;11:598–603.

17. Franklin KBJ, Paxinos G. The mouse brain in stereotaxic coordinates.

18. Lois-Milevicich J, Medina C, Fuente V de la. Statistical Analysis of Fear Conditioning Data Using Mixed Models: Methods and Applications. 2023. 2023. https://doi.org/10.31234/osf.io/pzxa6.
